# Aldh2 is a lineage-specific metabolic gatekeeper in melanocyte stem cells

**DOI:** 10.1242/dev.200277

**Published:** 2022-05-19

**Authors:** Hannah Brunsdon, Alessandro Brombin, Samuel Peterson, John H. Postlethwait, E. Elizabeth Patton

**Affiliations:** 1 MRC Human Genetics Unit, Institute of Genetics and Cancer, The University of Edinburgh, Western General Hospital Campus, Crewe Road, Edinburgh EH4 2XU, UK; 2 Cancer Research UK Scotland Centre, Institute of Genetics and Cancer, The University of Edinburgh, Western General Hospital Campus, Crewe Road, Edinburgh EH4 2XU, UK; 3 Institute of Neuroscience, University of Oregon, Eugene, OR 97403, USA

**Keywords:** Melanocyte stem cell, Regeneration, Aldh2, Formaldehyde, Metabolism, Purines

## Abstract

Melanocyte stem cells (McSCs) in zebrafish serve as an on-demand source of melanocytes during growth and regeneration, but metabolic programs associated with their activation and regenerative processes are not well known. Here, using live imaging coupled with scRNA-sequencing, we discovered that, during regeneration, quiescent McSCs activate a dormant embryonic neural crest transcriptional program followed by an aldehyde dehydrogenase (Aldh) 2 metabolic switch to generate progeny. Unexpectedly, although ALDH2 is well known for its aldehyde-clearing mechanisms, we find that, in regenerating McSCs, Aldh2 activity is required to generate formate – the one-carbon (1C) building block for nucleotide biosynthesis – through formaldehyde metabolism. Consequently, we find that disrupting the 1C cycle with low doses of methotrexate causes melanocyte regeneration defects. In the absence of Aldh2, we find that purines are the metabolic end product sufficient for activated McSCs to generate progeny. Together, our work reveals McSCs undergo a two-step cell state transition during regeneration, and that the reaction products of Aldh2 enzymes have tissue-specific stem cell functions that meet metabolic demands in regeneration.

## Abstract (precis)

**Summary:** In zebrafish melanocyte regeneration, quiescent McSCs respond by re-expressing a neural crest identity, followed by an Aldh2-dependent metabolic switch to generate progeny.

## INTRODUCTION

Melanocytes are pigment-producing cells that provide black-brown pigmentation in the hair, skin and eyes in the animal kingdom. Melanocytes can emerge directly from the neural crest during development, while other melanocytes come from melanocyte stem cells (McSCs), which are also neural crest derived and replenish the melanocyte populations in the adult (Mort et al., 2015). In mammals, distinct McSC populations serve as reservoirs for melanocytes that pigment the growing hair shaft, or for skin pigmentation in response to UV-irradiation or wound healing (Nishimura et al., 2005; Adameyko et al., 2009; Chou et al., 2013). In zebrafish, nerve-associated McSCs are an on-demand regenerative population at all stages, and the cell of origin for multiple pigment cell types as the zebrafish grows to become an adult (Budi et al., 2008, 2011; Dooley et al., 2013; Singh et al., 2016; Brombin et al., 2022). Imaging analysis over time as well as lineage-tracing studies show McSC progeny directly give rise to pigmented melanocytes (Dooley et al., 2013; Singh et al., 2016; Brombin et al., 2022). How McSCs respond to regenerative signals to generate melanocytes is a central question for adult stem cell biology, but also for melanoma pathogenesis, which is increasingly understood to re-activate and depend upon melanocyte lineage developmental programs in disease progression (White et al., 2011; Kaufman et al., 2016; Travnickova et al., 2019; Varum et al., 2019; Johansson et al., 2020; Marie et al., 2020; Baggiolini et al., 2021).

Zebrafish are uniquely poised for studying stem cells due to their genetic tractability and amenability to advanced imaging, enabling the intricacies of stem cell and developmental lineages to be followed at the single cell resolution in living animals (Kelsh et al., 1996; Owen et al., 2020; Travnickova and Patton, 2021). During zebrafish embryonic development, melanocytes that originate directly from the neural crest generate stripes along the body (Kelsh and Barsh, 2011). McSCs that reside at the dorsal root ganglion (DRG) stem cell niche are also established during embryogenesis, are multi-potent, and give rise to glia and multiple pigment cell types that contribute to the adult pigmentation pattern and serve as a source for melanocytes in regeneration (Budi et al., 2008, 2011; Hultman et al., 2009; Johnson et al., 2011; Kelsh and Barsh, 2011; Dooley et al., 2013; Singh et al., 2016; Irion and Nusslein-Volhard, 2019; Brombin et al., 2022).

**Fig. 1.**
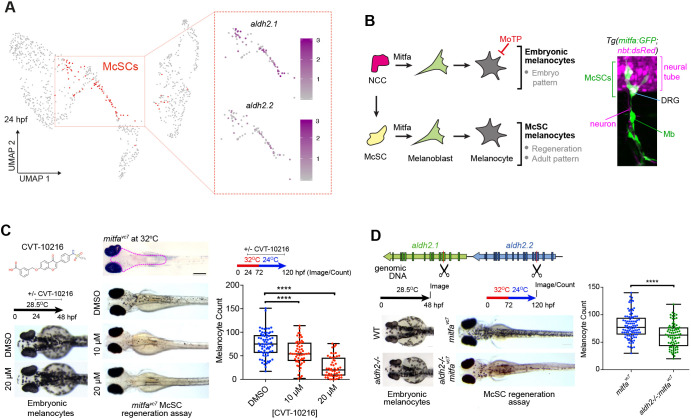
**Lineage-specific requirement for Aldh2 in melanocyte regeneration.** (A) UMAP of scRNA-seq data derived from 24 hpf embryos (Brombin et al., 2022) with McSCs in red. Feature plots of these isolated McSCs showing log_2_ expression of *aldh2* paralogs with colour change from grey (negative) to purple. (B) Schematic of the melanocyte lineages in zebrafish development with confocal *z*-stacks depicting McSCs expressing *mitfa:GFP* located at the dorsal root ganglia (DRG) and melanoblasts (Mb) on the motor neurons. Neural tube and DRG are marked by *nbt:dsRed* expression. (C) Representative images of wild-type embryos treated with or without CVT-10216 during development (embryonic melanocytes) or in an McSC regeneration assay. Regenerated melanocytes were quantified within a consistent region delineated by the magenta dotted line on the non-regenerating control embryo (top). One data point plotted per embryo; boxes indicate median and quartiles; whiskers span minimum to maximum values. Scale bar: 500 µm. ****P<0.0001 (one-way ANOVA with Tukey's multiple comparisons test). Four experimental replicates. (D) Schematic of CRISPR-Cas9 strategy to target *aldh2.1* and *aldh2.2* with excision site between Cas9 cut sites (scissor symbols; see Fig. S1). Wild-type or *aldh2^−/−^* embryos in normal development or a McSC regeneration assay is shown. ****P<0.0001. An unpaired two-tailed *t*-test was performed to calculate statistical significance. One data point is plotted per embryo; boxes indicate median and quartiles; whiskers span minimum to maximum values; three experimental replicates.

Recently, we identified an ErbB-dependent developmental *tfap2b^+^* McSC population that we found to be distinct within neural crest and pigment cell lineages, and which lineage-tracing analysis showed gave rise to all three zebrafish pigment cell types, including melanocytes, as well as nerve-associated cells (Brombin et al., 2022). Among the members of the zebrafish aldehyde dehydrogenase (Aldh) 1 and 2 enzyme family, which are well conserved with analogous human enzymes, we found *aldh2* gene paralogs were specifically expressed in these *tfap2b^+^* McSCs (Fig. 1A; Fig. S1). Aldehyde-processing enzymes are viewed as essential clearing agents that rapidly deactivate harmful aldehydes, and also as markers of somatic and cancer stem cell populations (O'Brien et al., 2005; Marcato et al., 2011; Pontel et al., 2015; Garaycoechea et al., 2018). In the bone marrow, two specific enzymes, aldehyde dehydrogenase (ALDH) 2 and alcohol dehydrogenase (ADH) 5, protect hematopoietic stem cells (HSCs) from endogenous formaldehyde accumulation and toxicity (Dingler et al., 2020; Nakamura et al., 2020; Oka et al., 2020; Jung and Smogorzewska, 2021; Mu et al., 2021). The importance of aldehyde detoxification in human biology is exemplified by the genetic variants of *ALDH2* in the human population, such as the single nucleotide polymorphism r671 in *ALDH2* (c.1510G>A; p.E504K; ALDH2*2), which confers loss of function in 560 million people, mainly of East Asian origin (Chen et al., 2014, 2020). Carriers of the r671 *ALDH2* polymorphism can experience adverse reactions to acetaldehyde from exogenous alcohol consumption and are at risk for a range of diseases, including osteoporosis, cardiovascular disease, neurodegeneration and Fanconi anaemia (Harada et al., 1981; Brooks et al., 2009; Hiura et al., 2010; Takeuchi et al., 2012; Matsuo et al., 2013; Masaoka et al., 2016; Chang et al., 2017).

Much of the toxicity from aldehydes can be attributed to metabolites such as acetaldehyde and formaldehyde, which cause mutations and chromosomal rearrangements by direct damage to DNA (Pontel et al., 2015; Garaycoechea et al., 2018). Recent work shows that a two-tier protection mechanism in cells defends against aldehyde-induced DNA crosslinks: first, aldehydes are cleared by enzymes, such as ALDH2 and ADH5; and second, replication-coupled DNA damage response pathways repair crosslinks and remove adducts (Langevin et al., 2011; Rosado et al., 2011; Garaycoechea et al., 2012, 2018; Pontel et al., 2015; Hodskinson et al., 2020). These studies emphasize the nature of aldehyde toxicity and homeostatic clearance, primarily investigated in the hematopoietic stem cell compartment. However, other work proposes more varied roles for aldehydes, namely that by-products generated by aldehyde detoxification enzyme reactions also sustain essential downstream cellular metabolic processes (Jacobson and Bernofsky, 1974; Bae et al., 2017; Burgos-Barragan et al., 2017). What is yet unknown is how the reaction products of aldehyde metabolism by ALDH2 contribute to the physiology of specific cells and tissues in processes other than toxicity. Here, we discover a new requirement for Aldh2-dependent metabolism in activated McSCs during regeneration.

## RESULTS

### A lineage-specific function for Aldh2 in melanocyte regeneration

To learn how ALDH2 functions in stem cells other than HSCs and in an intact animal, we set out to study the zebrafish McSC population in melanocyte regeneration. We employed the ALDH2 inhibitor (ALDH2i) CVT-10216 in a melanocyte regeneration assay that is dependent on a temperature sensitive splicing defect of the master melanocyte transcription factor MITF (*mitfa^vc7^*) (Johnson et al., 2011; Zeng et al., 2015). In wild-type embryos, neural crest-derived embryonic melanocytes pigment the epidermis during the first 72 h of development before McSCs are activated. The *mitfa^vc7^* regeneration model allows us to bypass embryonic pigmentation by growing *mitfa^vc7^* embryos at higher temperatures (such that *mitfa* is spliced incorrectly) to deplete Mitfa activity during this 72 h period. After this, melanocyte regeneration can be activated from McSCs in *mitfa^vc7^* embryos by lowering the water temperature to a level permissive for correct splicing of *mitfa*, thereby restoring its activity and allowing melanocytes to regenerate from McSCs over a period of 48 h (Johnson et al., 2011) (Fig. 1B). In zebrafish embryos grown in the presence of CVT-10216, we did not detect any discernible effects on embryonic melanocyte development. However, melanocyte regeneration from McSCs was significantly delayed in ALDH2i-treated embryos, indicating that Aldh2 has a lineage-specific function in McSCs (Fig. 1C; Fig. S1).

CVT-10216 is reported to have a >40-fold selectivity for ALDH2 over other ALDH enzymes (Chen et al., 2014); however, to confirm this specificity in zebrafish, we generated an *aldh2.1/aldh2.2* double mutant line by CRISPR-Cas9, henceforth referred to as *aldh2^−/−^*. The genetic similarity between these two paralogs made generating specific *aldh2* mutants difficult, so we created a double null mutant instead by designing guide RNAs to excise a large intergenic region between the tandem duplicate genes (Fig. S1). We confirmed loss of Aldh2 protein by western blotting (Fig. S1). In keeping with our ALDH2i experiments, *aldh2^−/−^* mutants generated embryonic melanocytes, yet were defective in melanocyte regeneration from the McSC compartment (Fig. 1D). We noticed that after multiple rounds of breeding, the melanocyte regeneration phenotype in our *aldh2^−/−^* mutants was lessened. This was coupled with transcriptional upregulation of other *aldh* enzyme family members, suggesting some plasticity in *aldh* expression in regeneration and the possibility of genetic compensation by other Aldh enzymes (El-Brolosy et al., 2019) (Fig. S1). To address this, we confirmed the *aldh2^−/−^* genetic mutant results in *aldh2.1* and *aldh2.2* knockdown experiments with translation-blocking morpholino oligonucleotides and once again showed that Aldh2 activity is specifically required in the McSC lineage, recapitulating the phenotype seen after Aldh2i (Fig. S1; Fig. 1C). Finally, we found that the embryonic melanocytes in *aldh2^−/−^* mutants were defective for the dopaminergic camouflage response, a neuronally regulated innate behaviour, reflecting the function for Aldh2 in dopamine metabolism (Yao et al., 2010). This phenotype recapitulates our previous data with Daidzin, another ALDH2i, and provides confidence that the *aldh2^−/−^* mutants are defective for Aldh2 activity (Zhou et al., 2012) (Fig. S1).

### Live imaging captures the McSC requirement for Aldh2 to generate progeny

**Fig. 2.**
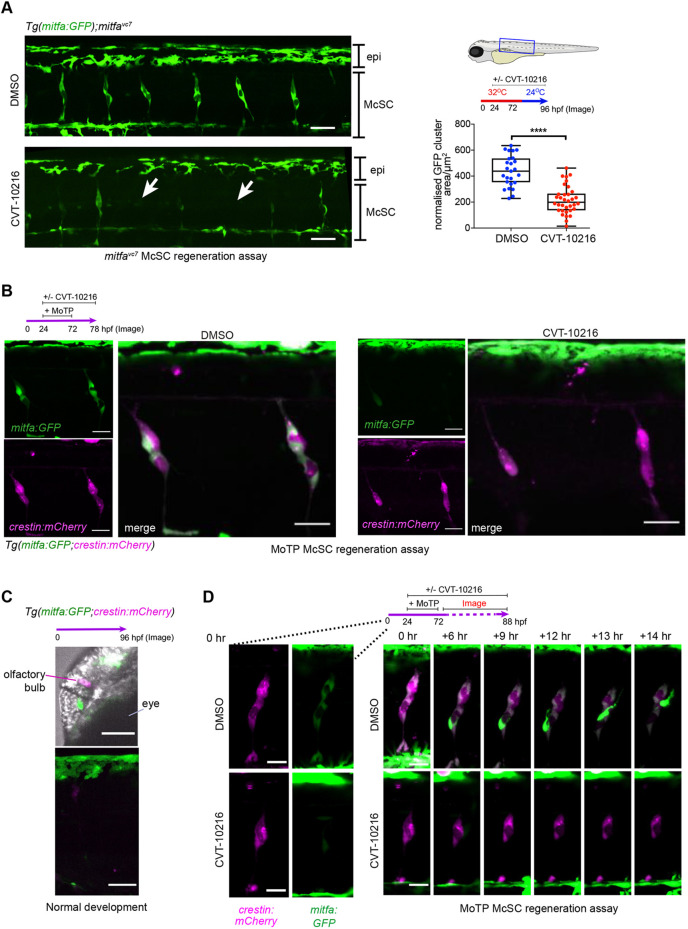
**Live imaging captures the McSC requirement for Aldh2 to generate progeny.** (A) An ALDH2 inhibitor (CVT-10216) causes loss of *mitfa:GFP* expression in McSCs, while dorsal stripe epithelial (epi) *GFP^+^* melanoblasts remain. Representative confocal stack images of McSCs at the niche after 24 h regeneration with or without CVT-10216 treatment. The average *mitfa:GFP* niche area μm^2^/somite was quantified per embryo (one data point) Boxes indicate median and quartiles; whiskers span minimum to maximum values. McSCs with very low to no GFP signal are indicated with arrows. Scale bars: 50 μm, three experimental replicates.****P<0.0001 (unpaired, two-tailed *t*-test). (B) McSCs maintain neural crest identity when treated with an ALDH2 inhibitor (CVT-10216). Confocal stack images of McSC niches in CVT-10216-treated *Tg(mitfa:GFP;crestin:mCherry)* embryos after 6 h washout of MoTP. Two experimental replicates, five or more embryos used per condition, representative images shown. Scale bars: 50 µm. (C) 96 hpf non-regenerating *Tg(mitfa:GFP;crestin:mCherry)* embryos (same age as B) still express *crestin:mCherry* in the olfactory bulb and *mitfa:GFP* in embryonic epithelial melanoblasts [labelled in head (top) and trunk (bottom)], but no longer express these transgenes in McSC niches. Representative images of three embryos are shown. Scale bars: 50 µm. (D) Time-lapse stills of individual regenerating McSCs at the niches. *Tg(mitfa:GFP; crestin:mCherry)* embryos with or without CVT-1016 were imaged from 2 h post-MoTP washout. In a control embryo, an McSC undergoes cell division and a new *mitfa:GFP*-high cell migrates upwards towards the epidermis (see Movie 3). In a CVT-10216-treated embryo, *mitfa:GFP* expression is absent and migration is not observed (see Movie 4). Scale bars: 20 µm.

To investigate whether Aldh2 activity impacts directly upon McSCs, we employed a *Tg(mitfa:GFP)* transgenic line that was previously shown to mark McSCs (Dooley et al., 2013; Brombin et al., 2022). Following ALDH2i treatment in regenerating embryos, we observed a significant reduction in GFP^+^ McSCs in the niche (Fig. 2A). One interpretation of this result is that McSCs are depleted in the absence of Aldh2. Alternatively, McSCs may be present but expressing only low *mitfa:GFP* under conditions of ALDH2 inhibition.

In the earliest stages of embryonic development, McSCs that emerge from the neural crest maintain a neural crest identity at the niche, but lose this identity by day 3 (Brombin et al., 2022). Given our results in ALDH2i-treated regenerating embryos, we postulated that regenerative (activated) McSCs would re-express neural crest identity markers in addition to *mitfa*. To assess this hypothesis, we employed a double-transgenic line *Tg(mitfa:GFP; crestin:mCherry)* in which *mCherry* is expressed from the promoter of the neural crest gene *crestin* (Kaufman et al., 2016; Brombin et al., 2022), and applied this to a second independent regeneration assay. In this assay, the pro-drug MoTP kills differentiated embryonic melanocytes, and melanocytes are regenerated from the McSC compartment (Fig. 1B) (Yang and Johnson, 2006). Following MoTP washout, McSCs expressed both mCherry and GFP in control animals (Fig. 2B)*.* McSCs were not detectable in non-regenerating embryos (without MoTP) (Fig. 2C). In regenerating embryos, the intensity of GFP was heterogeneous between McSC clusters, but all McSCs expressed mCherry, indicating that McSCs re-express a neural crest identity in regeneration (Fig. 2B). Upon ALDH2i treatment, and as seen in Fig. 2A, we again observed a specific and strong reduction of GFP in McSCs, with mCherry^+^ McSCs still being clearly visible. Imaging niches at a higher magnification revealed a significant reduction in *mitfa:GFP* cells within McSC niches (Fig. 2B; Fig. S2; Movies 1, 2). Thus, McSCs re-express a neural crest marker during regeneration and require Aldh2 to increase expression of *mitfa* and generate melanoblasts.

Using live confocal imaging of McSCs to capture this process over time, we performed an MoTP regeneration assay and observed cells expressing high levels of *mitfa:GFP* emerging from McSCs and migrating dorsally in control embryos (Fig. 2D; Movie 3). In contrast, the McSC niches in ALDH2i-treated embryos had little discernible cell movement, with very little *mitfa:GFP* expression (Fig. 2D; Movie 4). Taken together, these data show that there are at least two distinct cell states within the regenerative McSC niche (*mitfa-*low and *mitfa-*high) and that Aldh2 is required for activated McSCs to increase *mitfa* expression and generate migratory progeny.

### Aldh2, but not Adh5, is required for formaldehyde metabolism in McSCs

**Fig. 3.**
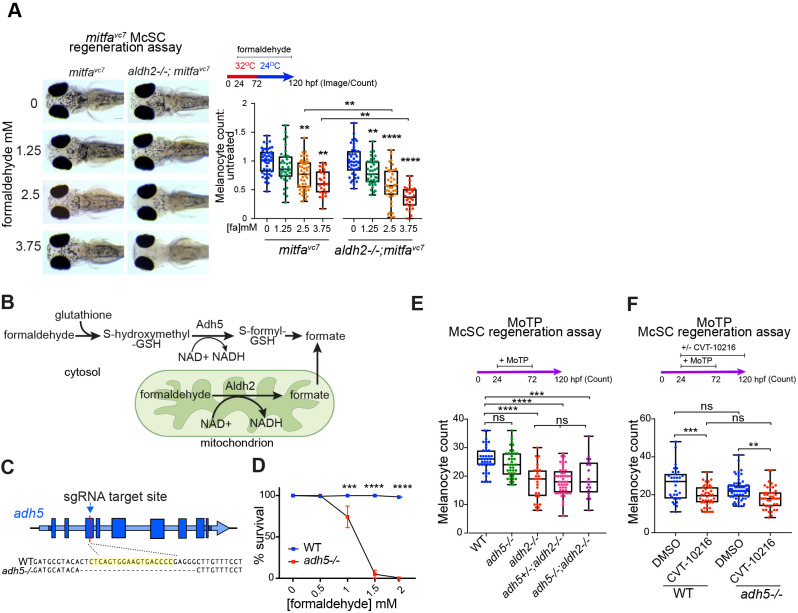
**McSCs require Aldh2, but not Adh5, for formaldehyde metabolism.** (A) Melanocyte regeneration is sensitive to formaldehyde and this effect is stronger in *aldh2^−/−^* mutants. Images and quantification of melanocytes in zebrafish embryos in a *mitfa^vc7^* regeneration assay. Melanocyte counts were normalised to the mean of the respective control, each dot represents a single embryo; boxes indicate median and quartiles; whiskers span minimum to maximum values; three experimental replicates. **P<0.0021, ****P<0.0001 (one-way ANOVA with Tukey's multiple comparisons). (B) Schematic diagram of formaldehyde metabolism by Adh5 (cytosol) and Aldh2 (mitochondria). (C) Schematic diagram showing the *adh5^−/−^* CRISPR-Cas9 mutant, with sgRNA target site in exon 3 and alignment to wild-type sequence showing a deletion of 25 bp. (D) Sensitivity of *adh5^−/−^* embryos to increasing concentrations of formaldehyde from 24 hpf for 24 h, and surviving embryos quantified. Five experimental replicates, 20 embryos per condition. ***P<0.0002, ****P<0.0001 (two way ANOVA with Sidak's multiple comparisons). Data are mean±s.e.m. (E) MoTP regeneration assay on *aldh2^−/−^*, *adh5^−/−^* mutant embryos and embryos from an incross of *adh5^+/−^; aldh2^−/−^* fish (embryos genotyped after counting). One data point plotted per embryo; boxes indicate median and quartiles; whiskers span minimum to maximum values; three experimental replicates. ***P<0.0002, ****P<0.0001; ns, not significant (one-way ANOVA with Tukey's multiple comparisons). (F) MoTP regeneration assay on wild type and *adh5^−/−^* mutants treated with or without CVT-10216. One data point plotted per embryo; boxes indicate median and quartiles; whiskers span minimum to maximum values; three experimental replicates. ***P<0.0002, **P<0.0021; ns, not significant (one-way ANOVA with Tukey's multiple comparisons).

To elucidate the mechanism by which Aldh2 affects transitions between cell states in McSCs, we sought to identify its substrate. We reasoned that aldehyde substrates in melanocyte regeneration would be toxic if supplied in excess, and that toxicity would increase in *aldh2^−/−^* mutant embryos. Therefore, we screened known ALDH2 substrates for sensitivity in zebrafish development overall and specifically in the context of melanocyte regeneration (Table 1; Fig. S2). We found that *aldh2^−/−^* embryos were resistant to acetaldehyde and propionaldehyde, suggesting an unexpected plasticity in response to these aldehydes. *aldh2^−/−^* mutants were sensitized to 4-HNE, but this was not specific to the McSC lineage (Fig. S2). Importantly, *aldh2^−/−^* embryos were sensitive to formaldehyde and, notably, to low doses of exogenous formaldehyde (that had no other apparent effect on the fish) impaired melanocyte regeneration. This response was significantly stronger in *aldh2^−/−^* mutants compared with controls (Fig. 3A; Fig. S2). These data indicate that formaldehyde, but not other aldehydes, is an important Aldh2 substrate in the McSC compartment.

**Table 1.**
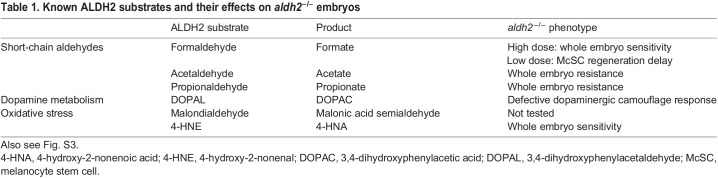
Known ALDH2 substrates and their effects on *aldh2^−/−^* embryos

Recent studies show that ALDH2 and ADH5 function together to clear endogenous formaldehyde during HSC differentiation to prevent immune depletion in mouse and induced pluripotent stem cells (iPSCs), as well as in individuals with biallelic *ALDH2* and *ADH5* mutations (Dingler et al., 2020; Oka et al., 2020; Shen et al., 2020; Mu et al., 2021) (Fig. 3B). Mice lacking both ALDH2 and ADH5 develop leukaemia and have shorter lifespans, and, despite active DNA repair, bone marrow-derived progenitors acquire a formaldehyde-associated mutation signature that resembles human cancer mutation signatures associated with aging (Dingler et al., 2020). To address whether Adh5 can function in melanocyte regeneration and compensate for Aldh2, we generated an *adh5^−/−^* mutant line by CRISPR-Cas9 (Fig. 3C). We found that the *adh5^−/−^* mutant was highly sensitive to exogenous formaldehyde treatment, indicating that, as in mammals, formaldehyde is an Adh5 substrate in zebrafish (Fig. 3D). However, *adh5* loss had no effect on melanocyte regeneration and did not enhance the regeneration defects in *aldh2^−/−^* mutants or ALDH2i-treated embryos (Fig. 3E,F). Thus, despite the shared formaldehyde substrate and conservation across species, Aldh2 has a unique function for formaldehyde metabolism in McSC differentiation and Adh5 does not compensate for Aldh2 in this cell lineage.

### scRNA-sequencing reveals Aldh2 is a metabolic gatekeeper for McSCs

**Fig. 4.**
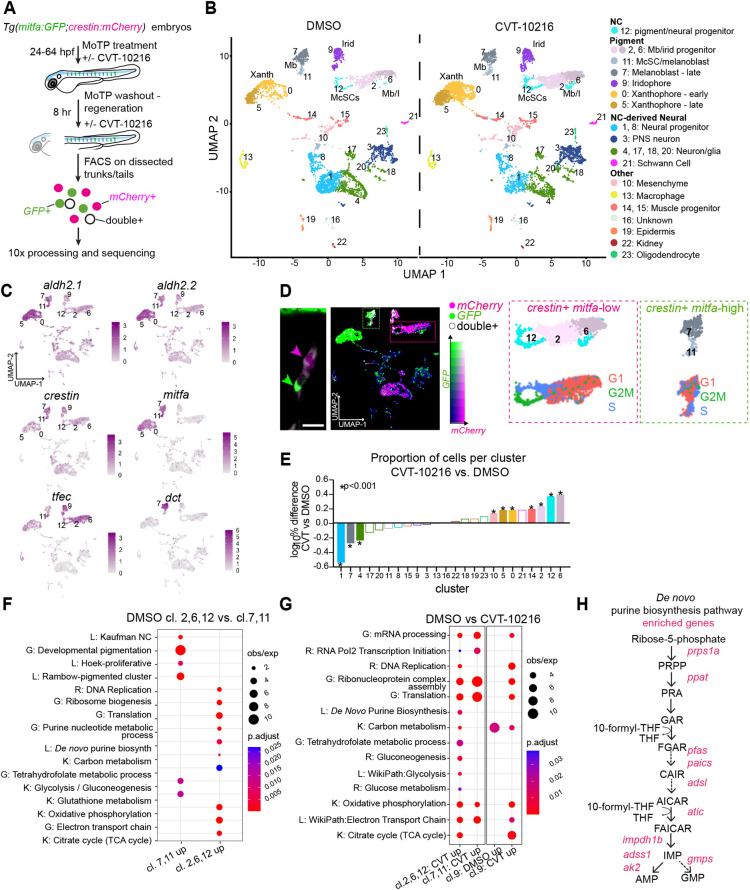
**scRNA-seq reveals an Aldh2 metabolic gatekeeper function.** (A) Experimental design for the scRNA-seq experiment to capture the McSCs in regeneration. (B) UMAPs of *Tg(crestin:mCherry, mitfa:GFP)*-positive cells after clustering, split by drug treatment. Mb, melanoblasts; Xanth, xanthophores; Irid, iridophores. (C) UMAPs of both DMSO- and CVT-10216-treated cells with colour change from grey (negative) to purple based on log_2_ expression of *aldh2.1* and *aldh2.2* in pigment lineages compared with *crestin* (neural crest), *tfec* (melanophore/iridophore progenitors), *mitfa* (early melanoblasts) and *dct* (late melanoblasts). (D) Proposed relationship of imaged McSCs to scRNA-seq clusters, using an example niche from Fig. 2D (scale bar is 20 µm) and UMAP coloured by expression intensity of *mCherry* (magenta) and *GFP* (green), and cells in which both are expressed (white). We predict that *crestin^+^ mitfa-*high cells (green arrow/box) are represented in clusters 7 and 11, and *crestin^+^ mitfa-*low cells (magenta arrow/box) are represented in clusters 2, 6 and 12. UMAPs of these clusters (top) and their predicted cell cycle phase (bottom) are shown. (E) The proportion of total cells within each cluster compared between treatment conditions. The log_10_ percentage difference of numbers of cells in the CVT-10216-treated clusters compared with DMSO equivalents was plotted, with asterisks indicating a significant difference in proportions (Chi squared test). (F) Dot-plot of pathway analysis showing selection of significantly upregulated GO (G), KEGG (K), reactome (R) and literature-based (L) terms in clusters 2, 6 and 12 compared with 7 and 11, and vice versa. Dot size represents observed/expected ratio and colour indicates adjusted *P*-value (Benjamini–Hochberg test). (G) As in F, but showing significant enrichment of pathways in CVT-10216-treated cells relative to DMSO from clusters 2, 6 and 12 (*crestin^+^ mitfa-*low), clusters 7 and 11 (*crestin^+^ mitfa-high*), and cluster 9 (predicted iridophores). (H) Schematic diagram of *de novo* purine biosynthesis, with genes encoding enzymes significantly upregulated in the CVT-10216 dataset from G shown in red.

Thus far, we had visually captured activated McSCs uncoupled from emerging progeny, and discovered a novel role for Aldh2 in this process in metabolizing endogenous formaldehyde in these cells. Next, we went on to investigate the transcriptional signatures of these cell populations by single cell RNA-sequencing (scRNA-seq) to ascertain how they might be affected by Aldh2 deficiency. To this end, we designed a scRNA-seq analysis of a MoTP melanocyte regeneration experiment in which double transgenic *mitfa:GFP; crestin:mCherry* embryos were treated with DMSO or CVT-10216, and then GFP^+^, mCherry^+^ and double-positive cells were sorted together by FACS and processed for sequencing using the 10x Genomics protocol (Fig. 4A). We identified 24 clusters of transcriptionally distinct cell populations by comparing the top 30 variably expressed genes, generating uniform manifold approximation and projections (UMAPs) featuring expression of known lineage-defining NC genes, and mapping the cluster identities from two recent zebrafish scRNA publications onto our data (Saunders et al., 2019; Farnsworth et al., 2020) (Fig. 4B,C; Fig. S3; Tables S1, S2).

As *crestin:mCherry* is expressed in a wide range of neural crest-derived cell populations (Kaufman et al., 2016), we captured both pigment cell lineages and cells of the neural lineage. Clusters 7 and 11 expressed *crestin* and *mitfa*, with cluster 7 enriched for later stage melanoblast markers, such as *dct*. Cells in clusters 2, 6 and 12 expressed *crestin*, but low *mitfa*, and mapping previously published scRNA-seq datasets onto this cluster reveals they contained a mix of pigment and neural cell identity markers, consistent with stem cell identity (Farnsworth et al., 2020; Brombin et al., 2022). Upon closer analyses of pigment cell clusters, we found that a subset of cluster 11 also shared these characteristics, suggesting that these are also McSCs (Fig. S3). *aldh2.2* and *aldh2.1* were expressed across multiple pigment cell clusters, including McSCs and melanoblasts (Fig. 4C). Relating the above cluster identities to our imaging analyses, we propose that the *crestin^+^ mitfa-*low McSCs are within clusters 2, 6 and 12, and that the *crestin^+^ mitfa-*high McSCs and progeny (and any remaining embryonic melanoblasts) are within clusters 7 and 11 (Fig. 4D)*.* The predicted cell cycle phase shows clusters 11 (*mitfa-high*) and 12 (*mitfa-low*) to be in S and G2/M, and may reflect the cycling McSCs we observe during regeneration (Figs 2D and 4D).

Next, we analysed the dataset by drug treatment condition. Overall, we found that Aldh2 inhibition did not substantially change cell or cluster identity (Fig. 4B). However, when comparing the numbers of cells within each cluster as a percentage of the total cell number per treatment condition, the proportions of cells within some clusters differed significantly (Fig. 4E). Specifically, we detected a higher proportion of *crestin^+^ mitfa-*low cells (clusters 2,6,12) and a lower proportion of *crestin^+^ mitfa*-high cells (cluster 7) after ALDH2i. This population shift is consistent with our imaging experiments, in which we detected fewer *mitfa:GFP*-expressing cells at the McSC niche (Fig. 2A,B,D), and is suggestive of a block in McSC differentiation.

To understand the physiological and mechanistic implications of the Aldh2-dependent *mitfa-*high to *mitfa-*low McSC transition, we performed differential expression analysis with the control dataset between *crestin^+^ mitfa-*low cells and *crestin^+^ mitfa-*high cells (Table S3). *mitfa-*high cells (clusters 7,11) were enriched for pigmentation programs and melanoma-related terms, whereas *mitfa-*low cells (clusters 2,6,12) were enriched for essential metabolic pathways, including the 1C (THF) cycle, the TCA cycle and *de novo* purine biosynthesis (Fig. 4F), suggesting that regenerative McSCs have metabolic requirements distinct from those of melanoblasts.

Next, to understand why McSCs require Aldh2 activity to generate progeny, we performed differential expression analyses between controls and ALDH2i-treated cell populations (Fig. 4G; Tables S4-S6). Within the ALDH2i-treated *crestin^+^ mitfa-*low cell population, *de novo* purine synthesis was again significantly upregulated (Fig. 4G,H; Fig. S3), suggesting that McSCs ‘blocked’ by ALDH2i are starved of purines. We found no ALDH2i-dependent change in *de novo* purine synthesis or glucose metabolism genes in cells from either clusters 7 or 11, or in another pigment cell cluster requiring purine synthesis for pigmentation (cluster 9; iridophores) (Ng et al., 2009). Therefore, this pattern was specific to *crestin^+^ mitfa-*low cells and not a general effect of drug treatment. Taken together, these analyses support a mechanism in which regenerative McSCs require Aldh2 for metabolic rewiring in order to generate progeny.

### Formate, the reaction product of Aldh2-dependent formaldehyde metabolism, promotes McSC transitions

**Fig. 5.**
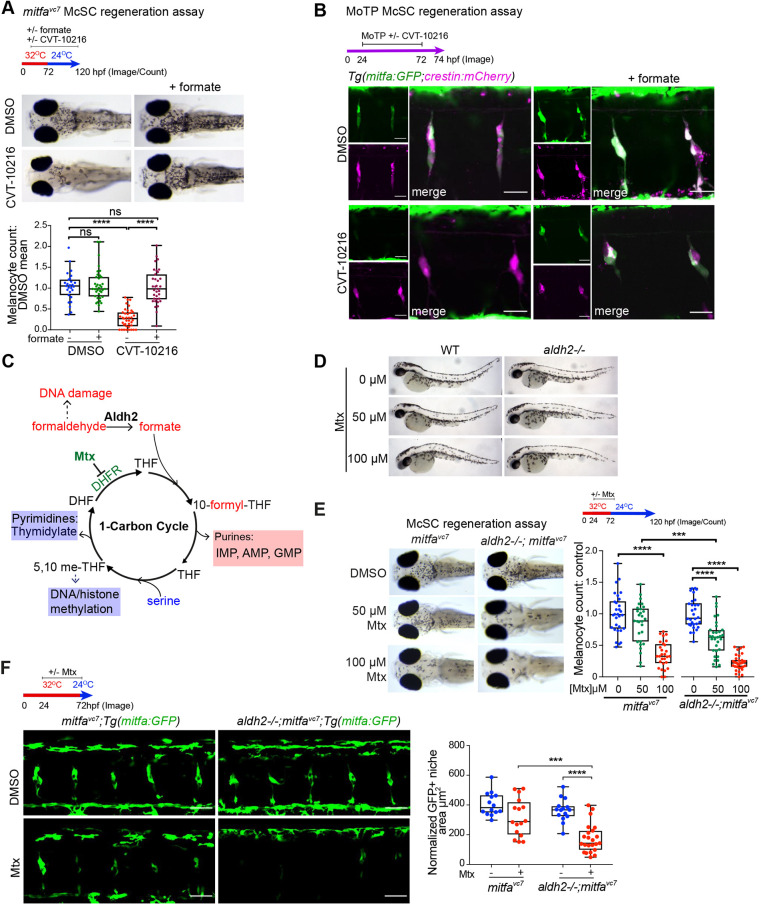
**The Aldh2 metabolic reaction product, formate, promotes McSC-derived progeny.** (A) Representative images of a regeneration assay where control or CVT-10216-treated embryos were supplemented with 25 mM sodium formate. ****P<0.0001; ns, not significant. Kruskal–Wallis test with Dunn's multiple comparisons. One data point per embryo; boxes indicate median and quartiles; whiskers span minimum to maximum values; three experimental replicates. (B) A MoTP assay on *Tg(mitfa:GFP;crestin:mCherry)* embryos treated with or without CVT-10216, and with or without 25 mM sodium formate from 24 hpf. MoTP was washed out at 72 hpf, and embryos imaged confocally at 74 hpf. Two experimental replicates, more than five embryos imaged per replicate. Scale bars: 25 µm. Single channel images of *crestin:mCherry* expression (magenta) and *mitfa:GFP* expression (green) are shown alongside merged channels. (C) Schematic of 1C metabolism and proposed function for Aldh2 formate supply through formaldehyde metabolism (based on Burgos-Barragan et al., 2017). Tetrahydrofolate (THF) combines with formate to make 10-formyl-THF, which provides two carbons to make purine nucleosides. (D) Mtx treatment has no effect on embryonic melanocytes. Zebrafish embryos (wild type and *aldh2^−/−^*) treated with or without Mtx at 24 hpf for 48 h. *n*=3. (E) Representative images of control and *aldh2^−/−^* mutants with or without Mtx treatment in a *mitfa^vc7^* regeneration assay. The melanocyte count at each dose was normalised to its respective DMSO control. ***P<0.0002, ****P<0.0001 (one-way ANOVA performed with Tukey's multiple comparisons test). One data point plotted per embryo; boxes indicate median and quartiles; whiskers span minimum to maximum values; three experimental replicates. (F) Confocal *z*-stacks of *mitfa:GFP* McSCs in a *mitfa^vc7^* regeneration assay, in control or *aldh2^−/−^* embryos treated with or without Mtx. Scale bars: 50 µm. Two experimental replicates; boxes indicate median and quartiles; whiskers span minimum to maximum values; more than five embryos imaged per repeat. Quantification of GFP^+^ niche area/somite of embryos treated with Mtx is shown. ***P<0.0002, ****P<0.0001 (one-way ANOVA with Tukey's multiple comparisons).

One explanation for the Aldh2-deficient regeneration phenotype is that accumulation of endogenous formaldehyde causes McSC toxicity. However, we believe this to be unlikely given our experimental data: (1) our observations while imaging over time showed no evidence of McSC disappearance; (2) following ALDH2i treatment, *crestin^+^ mitfa-*low McSCs were present in our scRNA-seq analysis, even at relatively higher numbers; and (3) the McSC block by ALDH2i treatment was reversible following washout (Fig. S1). These findings led us to hypothesize that the reaction products of formaldehyde metabolism are required for timely McSCs differentiation but not for survival. To test this hypothesis, we performed a regeneration assay in CVT-10216-treated embryos in the presence or absence of formate and found that formate supplementation fully restored melanocyte regeneration (Fig. 5A). At the cellular level, formate even fully rescued *crestin^+^ mitfa-*high McSCs at the niche site, while having no noticeable effect on *crestin:mCherry* expression (Fig. 5B). These results indicate that formate, an Aldh2-dependent reaction product, promotes McSCs to transition from a *mitfa-*low to *mitfa-*high state to generate progeny.

### McSCs require a functional 1C cycle

Formate is a carbon donor for the 1C cycle (Fig. 5C). We found the McSC metabolic switch identified here was reminiscent of cell state transitions reported for naïve to primed murine stem cells that depend on 1C cycling and nucleotide biosynthesis (Chandrasekaran et al., 2017), as well as formate overflow mechanisms that induce a metabolic shift from low to high adenine nucleotide levels in human cancer cell lines and mouse cancer models (Oizel et al., 2020). Indeed, 1C metabolism, compartmentalized within different cell types and organs, is becoming more broadly recognized as a physiological process impacting on cell states and associated with disease (Ducker and Rabinowitz, 2017). Taken together, our data suggest that regenerative McSCs depend on 1C cycling to transition from a neural crest to a melanoblast cell state.

To test this hypothesis, we used the dihydrofolate reductase inhibitor methotrexate (Mtx) to inhibit 1C metabolism (Fig. 5C-F). Mtx had no effect on the embryonic melanocyte lineage but its inhibitor function was easy to validate in zebrafish embryos; wild-type embryos treated with Mtx lack pigmentation in xanthophores and iridophores, both of which require functional 1C metabolism for pigment synthesis (Ng et al., 2009) (Fig. 5D; Fig. S4). In the McSC lineage, we found that Mtx treatment caused melanocyte regeneration defects that were significantly exacerbated in *aldh2^−/−^* mutants (Fig. 5E,F). These data indicate that zebrafish McSCs have metabolic requirements that require functional 1C metabolism.

### Aldh2-dependent formaldehyde metabolism meets the demand of McSCs for purines

**Fig. 6.**
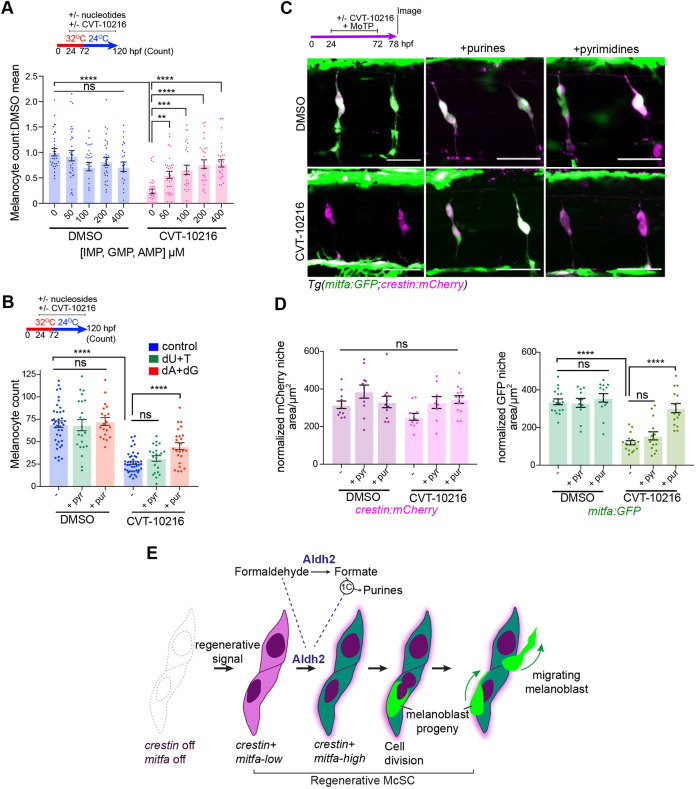
**Aldh2 meets the demand of McSCs for purines.** (A) Purine nucleotides rescue Aldh2-deficient melanocyte regeneration. Melanocyte regeneration assay in *mitfa^vc7^* embryos with or without CVT-10216 plus purine nucleotide cocktail. Melanocyte counts normalized to respective untreated controls. Each dot represents a single embryo, three experimental replicates. Data are mean±s.e.m. **P<0.0021, ***P<0.0002, ****P<0.0001; ns, not significant (one-way ANOVA with Tukey's multiple comparisons). (B) Purine, but not pyrimidine nucleosides, rescues Aldh2-deficient melanocyte regeneration. Melanocyte regeneration assay on *mitfa^vc7^* embryos with or without CVT-10216 and supplemented with deoxyadenosine (dA), deoxguanosine (dG) or deoxyuridine (dU) or thymidine (T) nucleosides (200 µM). Each datapoint represents a single embryo, three experimental replicates. ****P<0.0001; ns, not significant (one-way ANOVA with Tukey's multiple comparisons). Data are mean±s.e.m. (C) Purine nucleotides rescue McSC differentiation in ALDH2i-treated embryos. Representative confocal *z*-stacks of *Tg(mitfa:GFP;crestin:mCherry)* embryos treated with MoTP with or without CVT-12016, as well as 400 µM AMP/GMP purine nucleotides, or 400 µM UMP/thymidine pyrimidine nucleotides. Two experimental replicates, more than five embryos per condition. (D) Quantification of *crestin:mCherry* and *mitfa:GFP* niche areas from C. Each dot represents the sum of the GFP or mCherry niche area/ number of somites in view in one embryo. ****P<0.0001; ns, not significant (one-way ANOVA with Tukey's multiple comparisons). Data are mean±s.e.m. (E) Proposed model for Aldh2-mediated control of the McSC lineage. Regenerating McSCs start expressing *crestin* and low levels of *mitfa*. Next, McSCs increase their metabolic demands for purine nucleotides to express high levels of *mitfa* and generate progeny. This metabolic demand is met by Aldh2 metabolizing endogenous formaldehyde into formate, which is then used in the 1C cycle to fuel production of purine nucleotides. McSCs undergo cell division to generate progeny, which migrate away from the niche to the epidermis. ALDH2i (CVT-10216) delays the progression of the activated McSCs to generate progeny in regeneration.

Given the upregulation of *de novo* purine metabolism genes in McSCs and their dependency on 1C metabolism, we next set out to examine purine nucleotide supplementation in regeneration. In the presence of ALDH2i, we found that exogenously provided purine nucleotides rescued the melanocyte regeneration defect in a dose-dependent manner (Fig. 6A). This effect was not simply a consequence of providing embryos with an additional energy source in the form of ATP, because purine ribonucleosides were also capable of rescuing melanocyte regeneration (Fig. 6B). However, pyrimidine supplementation did not rescue melanocyte regeneration, demonstrating that this effect does not reflect a general requirement for all nucleotides. Next, we explored the specificity of this rescue using confocal imaging and found that purine, but not pyrimidine, supplementation selectively rescued *mitfa:GFP* expression at the McSC niche after ALDH2i treatment (Fig. 6C,D). Hence, McSCs have a specific requirement for Aldh2 to generate progeny, and the end product of Aldh2 formaldehyde metabolism in McSCs is purine nucleotides (Fig. 6E).

## DISCUSSION

Understanding McSC responses to regenerative signals is central to the search for druggable targets for regenerative medicine and melanoma therapies (Patton et al., 2021). Here, we coupled single cell RNA-sequencing with live imaging and chemical genetics in zebrafish McSCs to delineate how quiescent McSCs become activated and then transition to a proliferative state. By screening aldehyde substrates, we find melanocyte regeneration is sensitive to formaldehyde and is independent of *adh5*, and that the reaction product formate is sufficient to rescue Aldh2 deficiency. Thus, we identified an Aldh2-dependent mechanism exerting metabolic control of regeneration in McSCs, distinct from its aldehyde clearing mechanism. 8% of the world's population carry activity-reducing *ALDH2* mutations and the underlying disease mechanism is considered to be elevated cellular toxicity. Thus, identification of an ALDH2-dependent gatekeeper mechanism for a regenerative stem cell response may have important ramifications for carriers of inactivating ALDH2 variants.

We find that regenerative McSCs reactivate a neural crest identity, which is reminiscent of the neural crest and melanocyte developmental states that become reactivated in melanoma disease progression (White et al., 2011; Shakhova et al., 2012; Konieczkowski et al., 2014; Kaufman et al., 2016; Rambow et al., 2018; Travnickova et al., 2019; Varum et al., 2019; Diener and Sommer, 2020; Johansson et al., 2020; Marie et al., 2020). Although Dooley et al. (2013) detect *mitfa:GFP* expression at the niche throughout development, we consistently see a downregulation of *mitfa:GFP* expression in McSCs following establishment at the niche in non-regenerative conditions (Fig. 2; Brombin et al., 2022); these differences may possibly be due to differences in imaging parameters and/or transgene expression. We use a combination of *aldh2* genetic mutants, morpholino knockdown studies and a highly selective ALDH2 inhibitor to reveal a function for Aldh2 in McSC metabolism (Fig. 1; Fig. S1). Given the selective expression of *aldh2* enzymes in the McSC (Fig. S1) and the high selectivity of CVT-10216 for ALDH2 over other ALDH enzymes, Aldh2 is likely the primary target of CVT-10216 in the McSC context, although additional studies will be required to understand whether other ALDH enzymes are targets of CVT-10216 *in vivo*.

Notably, although all cells require nucleotides as fundamental building blocks, and for energy and signalling, the neural crest is especially sensitive to nucleotide depletion, which has direct metabolic consequences in rare disease and melanoma (Sporrij and Zon, 2021). For example, individuals with Miller syndrome, a rare genetic neurocristopathy affecting face and limb development, have mutations in dihydroorotate dehydrogenase (*DHODH*), the rate-limiting enzyme for pyrimidine *de novo* biosynthesis (Ng et al., 2010; Sporrij and Zon, 2021). In zebrafish, expression of a neural crest program defines melanoma initiation, and these cancers are sensitive to leflunomide, a DHODH inhibitor (White et al., 2011; Kaufman et al., 2016). Similarly, in mouse, a metabolic gene program driven by the transcription factor Yin Yang 1, a neural crest stem cell regulator, is essential for neural crest lineages, and its loss of function causes hypoplasia and prevents initiation of melanoma (Varum et al., 2019). In these contexts, nucleotide sensors may directly influence the transcriptional response, as we and others have shown for the neural crest and McSC differentiation (Johansson et al., 2020; Santoriello et al., 2020).

We were surprised to discover that regenerative McSCs have a select requirement for purine nucleotides (rather than pyrimidine nucleotides), findings that may point to purine nucleotide functions beyond transcription or DNA replication. For example, purine nucleotides have an ancient function as neurotransmitters that activate purinergic receptors, and as such can regulate neural stem and progenitor cells, and melanocyte-keratinocyte communication in human skin (Ulrich et al., 2012; Lee et al., 2019). Hence, purine nucleotides could facilitate McSC communication with DRG niche cells (of which we know very little) and with peripheral nerves that are used as migratory routes for melanoblast progenitors (Budi et al., 2011; Dooley et al., 2013). Given that neural crest and McSC programs re-emerge in melanoma, our findings may be relevant to understanding the metabolic reprogramming in melanomas, such as the dependency on folate metabolism during melanoma metastasis (Piskounova et al., 2015; Fischer et al., 2018).

How stem cells generate progeny is a fundamental question in regenerative medicine. Here, we show that Aldh2-dependent formaldehyde metabolism underlies McSCs metabolic demand for purines to generate progeny. Formaldehyde is abundant in the blood (>40 µM) and can arise from demethylation reactions from histones and nucleic acids (Dingler et al., 2020; Mu et al., 2021). While ALDH2 is often thought of as a protective enzyme, we find no evidence of McSC toxicity in zebrafish with defective Aldh2 activity. Based on our data in Fig. 3, we suggest that an unknown endogenous formaldehyde source is active in melanocyte regeneration. Conceptually, our work identifies an unanticipated lineage-specific requirement for Aldh2 in the supply of essential metabolites in McSCs. This could mean that, in individuals with inactivating mutations in ALDH2, both aldehyde cytotoxicity and depletion of aldehyde-derived metabolites could result in the clinical disease features.

## MATERIALS AND METHODS

### Data and code availability

scRNA-seq experiment data have been deposited in GEO under accession number GSE183868. Previously published sequencing data that were reanalysed here are available in GEO [GSE131136 (Saunders et al., 2019) and GSE178364 (Brombin et al., 2022)] and NCBI SRA [PRJNA564810 (Farnsworth et al., 2020)].

### Fish husbandry, fish lines

Zebrafish were maintained in accordance with UK Home Office regulations, UK Animals (Scientific Procedures) Act 1986, amended in 2013, and European Directive 2010/63/EU under project licences 70/8000 and P8F7F7E52. All experiments were approved by the Home Office and AWERB (University of Edinburgh Ethics Committee). Fish stocks used were: wild-type AB, *mitfa^vc7^* (Johnson et al., 2011; Zeng et al., 2015), *Tg(mitfa:GFP)* (Dooley et al., 2013), *Tg(crestin:mCherry)* (Kaufman et al., 2016), *aldh2^−/−^* (this study) and *adh5^−/−^* (this study). Combined transgenic and mutant lines were generated by crossing. Adult fish were maintained at ∼28.5°C under 14:10 light-dark cycles. Embryos were kept at either 24°C, 28.5°C or 32°C and staged according to the reference table provided by Kimmel and colleagues (1995).

### Genotyping

Whole embryos or fin clips from adult fish were genotyped by resuspending tissue in DirectPCR DNA-Tail solution (Viagen Biotech) and heating samples to 56°C for 2 h, then to 84°C for 20 min. Primers used for genotyping can be found in Table S7.

### CRISPR-Cas9 mutant line generation

sgRNAs (Table S7) were synthesized using the EnGen sgRNA Synthesis Kit, *S. pyogenes* (New England Biolabs) according to manufacturer's instructions. CRISPR-Cas9 knockout lines were generated as previously described (Sorlien et al., 2018). Briefly, 200 ng/μl sgRNAs targeting exon 3 of *aldh2.1* (GCCAGAGATGCCTTTAAGCT) and exon 3 of *aldh2.2* (GCCAGAGATGCCTTTAAGCT) were co-injected with Cas9 mRNA into zebrafish embryos at the one-cell stage. An allele was recovered that was the result of a large deletion between *aldh2.1* and *aldh2.2*, creating a gene fusion and single base-pair insertion at the fusion site. This introduced an adjacent frameshift mutation and premature stop codon.

200 ng/μl sgRNA targeting exon 3 of *adh5* (CTCAGTGGAAGTGACCCCGAG) was co-injected with recombinant 300 ng/μl Cas9 protein (SBI). These F0 fish were raised to adulthood, and outcrossed with wild-type fish to obtain progeny that were screened for presence of indels through PCR amplification of a 600 bp region surrounding the target site, and digestion of the amplicon using T7 endonuclease (New England Biolabs). Outcrossed F1 fish that contained a 25 bp deletion were isolated and raised to adulthood.

### Morpholino injection

Standard control morpholinos and translation blocking morpholinos were sourced from Genetools, based on previously published sequences for *aldh2.1* (ZDB-MRPHLNO-120517-2) and *aldh2.2* (ZDB-MRPHLNO-120517-3) (Ma et al., 2010). 2-6 ng of each morpholino was injected into sibling *mitfa^vc7^* embryos at the one- to two-cell stage.

### Imaging

Images of embryos immobilized with MS:222 and 1.5% LMP agarose were acquired using a 20×/0.75 lens on the multimodal Imaging Platform Dragonfly (Andor Technologies) equipped with 405, 445, 488, 514, 561, 640 and 680 nm lasers built on a Nikon Eclipse Ti-E inverted microscope body with Perfect focus system (Nikon Instruments). Data were collected in Spinning Disk 40 μm pinhole mode on the Zyla 4.2 sCMOS camera using a Bin of 1 and no frame averaging using Andor Fusion acquisition software. *Z* stacks were collected using the Nikon TiE focus drive. Multiple positions were collected using a Sigma-Koki Stage (Nikon Instruments). Data were visualized and analysed using Imaris (Oxford Instruments, v. 9.7.0) or Image J Fiji software (v. 1.53c).

Whole zebrafish embryos fixed in 4% PFA/PBST were imaged with a Leica MZFLIII fluorescence stereo microscope with a 1× objective fitted with a Qimaging Retiga Exi CCD camera (Qimaging). Image capture was performed using Micromanager (Version 1.4).

To quantify the area of *GFP* or *mCherry*-expressing cells within niches, homozygous *Tg(mitfa:GFP)* fish were outcrossed with non-fluorescent or *Tg(crestin:mCherry)* fish to obtain embryos with similar levels of transgene expression. The McSC compartment was imaged at the same magnification, within the same anatomical area, and with consistent laser power and other imaging settings between individual samples and biological replicates. In Fiji, a maximum projection z-stack of images was cropped to only include McSC compartment cells (typically containing six or seven compartments per image) and converted to a binary image. Consistent threshold settings were applied, and the total GFP^+^ area measured in pixels^2^ and divided by the number of somites visible in the field of view.

### Melanocyte regeneration assays

If using the *mitfa^vc7^* regeneration model line, embryos were kept in a 32°C incubator from 0 to 72 hpf to repress the developmental melanocyte lineage. Embryos were then moved to a 24°C incubator to allow regeneration over a period of 48 h. When using chemical methods for regeneration, 150 µM 4-(4-morpholinobutylthio)phenol (MoTP) (Sigma-Aldrich) was added to embryos kept at 28.5°C from 24 hpf onwards. MoTP was washed out to allow regeneration between 72 and 120 hpf. After fixation, embryos were imaged and melanocytes counted using the Image J CellCounter plug-in within a consistent dorsal area outlined in Fig. 1C. Embryos were imaged dorsally, and only in-focus dorsal surface melanocytes counted. For the anterior and posterior bounds, anatomical landmarks used include the anterior-most region of the head, but exclude any in-focus melanocytes around the mouth. Posteriorly, we counted until the point at which the yolk ‘pinches off’ as it meets the tail. This gave a uniform and wide area within which to count melanocytes consistently and gauge differences in number between drug treatments.

### Camouflage response assays

The camouflage response assay was performed as described previously (Zhou et al., 2012). 5 dpf wild-type or *aldh2^−/−^* mutant embryos were placed in darkness for 15 min to standardize their light exposure. These embryos were split into cohorts that were either placed under a lamp or kept in the dark for 1.5 h. The embryos were then moved to the opposite light condition for a further 45 min, during which time melanin dispersed or contracted depending on light exposure. This was repeated once or twice more when assessing the ability of the embryo to learn to adapt to changing light conditions. Afterwards, embryos were then briefly anaesthetized in MS-222 and fixed in 4% PFA. Embryos were imaged dorsally at a fixed magnification. Melanin coverage was measured with Image J Fiji, by outlining a predetermined region of the head, converting the image to an 8-bit binary image with a uniform threshold, and then measuring the area of black pixels.

### Small molecule inhibitor and rescue experiments

Unless otherwise stated, 10 μM CVT-10216 (Sigma-Aldrich) or equimolar dimethyl sulphoxide DMSO (Sigma-Aldrich) was added to embryos at 24 hpf after manual or pronase-assisted (Merck) dechorionation and refreshed every 24 h. Embryos were arrayed in six-well tissue culture plates with 10-15 embryos per well. For formate supplementation assays, 25 μM sodium formate (Merck) was added. For nucleotide supplementation assays, 400 μM of AMP, UMP, GMP, IMP or TMP (Merck) were added to embryos, or 200 μM of dA, dG, dU or T (Merck). 4-HNE (range of concentrations in ethanol) (Calbiochem) and Mtx (Merck) (range of concentrations in DMSO) were added at 24 hpf and refreshed every 24 h.

### Aldehyde treatments

Stock solutions of fresh acetaldehyde (Merck) and formaldehyde (VWR International) were made in a fume hood immediately before use. Various aldehyde concentrations were added to embryos kept in screw cap centrifuge tubes to limit aldehyde evaporation, and embryos scored for survival after 48 h.

### RNA extraction and RT-qPCR

Samples to be processed for RT-qPCR were collected at the required stage and frozen on dry ice. RNA was extracted from frozen tissues with the Qiagen RNeasy Mini kit according to manufacturer's instructions. RNA was quantified and quality checked using a Nanodrop 2000c (Thermo Scientific). 500 μg of RNA was used as input for Reverse Transcription using Superscript III reverse transcriptase (Invitrogen) and an Oligo(DT)_15_ primer (Promega). RT-qPCR was performed with Sybr Green Lightcycler Green I Master mix (Roche), using a Lightcycler 480 instrument and associated software. RT-qPCR primers (Table S7) were designed using Primer 3 Plus software to amplify ∼120 bp regions over exon-intron junctions. In the case of using RT-qPCR to detect *aldh2^−/−^* mutant transcripts, these regions were picked to be either in a predicted region of *aldh2.1* still present after the fusion event (primer site 1, exon 5 of *aldh2.1*, Fig. S1) or within a region of *aldh2.2* predicted to disappear after excision of the intergenic region between *aldh2.1* and *aldh2.2* (primer site 2, exon 13 of *aldh2.2*). β-actin was used as a housekeeping control. Gene expression fold changes were found using the ΔΔCt method.

### Western blotting

50 wild-type or *aldh2−/−* mutant embryos were processed for western blot analysis by deyolking and then homogenizing in RIPA buffer containing cOmplete Protease Inhibitor cocktail (Merck). After centrifugation, the protein content of the supernatant was measured with a BCA Protein Assay Kit (Thermo Fisher), and ∼10 μg of the protein was loaded onto a Mini-PROTEAN TGX Precast Gel (BioRad). Nitrocellulose membranes (BioRad) were blocked with 5% BSA in PBS-Tween and incubated with either anti-ALDH2 antibody (1:2000, 15310-0-1-AP, Proteintech) or anti-Histone H3 antibody (1:1000, ab10799, Abcam) overnight and then with IRDye 800CW donkey anti-rabbit IgG secondary antibody (1:10,000, RRID: AB_621848, Licor) or IRDye 680RD donkey anti-mouse IgG secondary antibody (1:10,000, RRID: AB_10953628, Licor), respectively, for 1 h. Blots were imaged using the Odyssey Infrared Imaging System and associated software (Licor).

### Single cell sequencing experimental setup and sequencing

24 hpf *Tg(mitfa:GFP; crestin:mCherry)* were divided into groups of ∼500 embryos and treated with MoTP, and co-treated with either 10 μM CVT-10216 or equimolar DMSO. At 64 hpf, MoTP was washed out, and embryos left to regenerate for 8 h. Embryos were anaesthetized in MS-222 and trunks dissected, and a cell suspension of each treatment condition obtained as previously described (Manoli and Driever, 2012). Samples were sorted on a FACSAria2 SORP instrument (BD Biosciences) as previously described (Brombin et al., 2022) but stage-matched non-fluorescent AB embryos also treated with MoTP were used as a control to enable gating of *mCherry* and *GFP* fluorescence. 10,000 GFP^+^, mCherry^+^ or double-positive cells per treatment condition were sorted together into 100 μl of 0.04% BSA/PBS and processed for the 10x protocol. Single cell libraries were prepared using the Chromium Single Cell 3′ GEM, Library & Gel Bead Kit v3 (10x Genomics).

The samples were sequenced on a Nextseq 2000 using a P2 flow cell on a 100 cycle run. ∼2.97 M reads passed quality check filters for CVT-10216 treated samples and ∼1.87 M reads passed quality check filters for DMSO-treated samples; however, due to the greater number of cells processed in the CVT-10216 sample, the mean reads per cell were fairly equal (37,405 for CVT versus 34,832 for DMSO).

### Bioinformatics analyses

*Aldh2.2* expression within developmental melanocytes was visualized using the recent scRNA-seq dataset of Brombin et al. (2022) (deposited in GEO under accession number GSE178364). For this study, FASTQ files were generated using CellRanger (v. 3.1.0, 10x Genomics) mkfastq function with default settings and -qc parameter, and aligned to the zebrafish STAR genome index using gene annotations from Ensembl GRCz11 release 94 with manually annotated entries for *GFP* and *mCherry*. Libraries were aggregated (CellRanger aggr pipeline) to generate a gene-barcode matrix. Gene matrices (13,360 total: DMSO, 5394; CVT, 7966), barcodes and features were uploaded to R (v. 4.0.5) and standard quality control filtering performed as previously described, to yield 4488 DMSO and 6795 CVT cells (Brombin et al., 2022). The dimensionality of the combined dataset was visualized with Elbow and JackStraw plots before running linear dimensional reduction. Louvain clustering was then performed using the FindNeighbors and FindClusters functions (dims=50, resolution=0.5) in Seurat (v. 4.0.3) (Hao et al., 2021). Data were projected onto 2D spaces using the same dimensions, using uniform manifold approximation and projection (UMAP). Cluster-specific genes were identified using Seurat, as previously described (Tables S1 and S2) (Brombin et al., 2022).

Cluster calling was performed as previously described (Brombin et al., 2022) and by making unbiased pairwise comparisons based on gene overdispersion against the GEO dataset GSE131136 (Saunders et al., 2019) and the NCBI SRA dataset PRJNA564810 (Farnsworth et al., 2020) and between the datasets presented in this paper as previously described (Brombin et al., 2022). Plots were generated either using Seurat or ggplot2 (v. 3.3.5) (Wickham, 2016). Prediction of cell cycle phase was performed with Seurat, using canonical cell cycle markers described previously (Tirosh et al., 2016).

For DE analyses, scRNA-seq data were first corrected for zero-inflated counts by using the ZINB-WaVE package (v. 1.12.0) with default parameters (Risso et al., 2019). The DEseq2 package (v. 1.30.1) (Love et al., 2014) was then used to generate gene lists of significantly (*P*.adj<0.05) upregulated and downregulated genes (raw data are in Tables S3-S6). Pathway analyses were performed as previously described (Travnickova et al., 2019). GSEA analysis was performed using GSEA software (v. 4.1.0) with gene lists generated through DeSeq2, using the ‘RunGSEAPreranked’ function. For phylogenetic analyses, FASTA sequences for human and zebrafish ALDH proteins were aligned with the online T-Coffee multiple sequence alignment, and the phylogeny feature used to construct a tree.

### Statistics

Statistical details of experiments and *n* numbers can be found in figure legends. Statistics and plots were generated using GraphPad Prism 7 (v. 7.0e) and R.

## Supplementary Material

Supplementary information

Reviewer comments
