# Supplementary material for: Aldh2 is a lineage-specific metabolic gatekeeper in melanocyte stem cells
Source: Development. 2022 May 19;149(10):dev200277. doi: 10.1242/dev.200277 (PMC9188749; doi:10.1242/dev.200277)
Supplement: Supplementary information [file develop-149-200277-s1.pdf]

Brunsdon *et al.*, Supplemental Figure S1

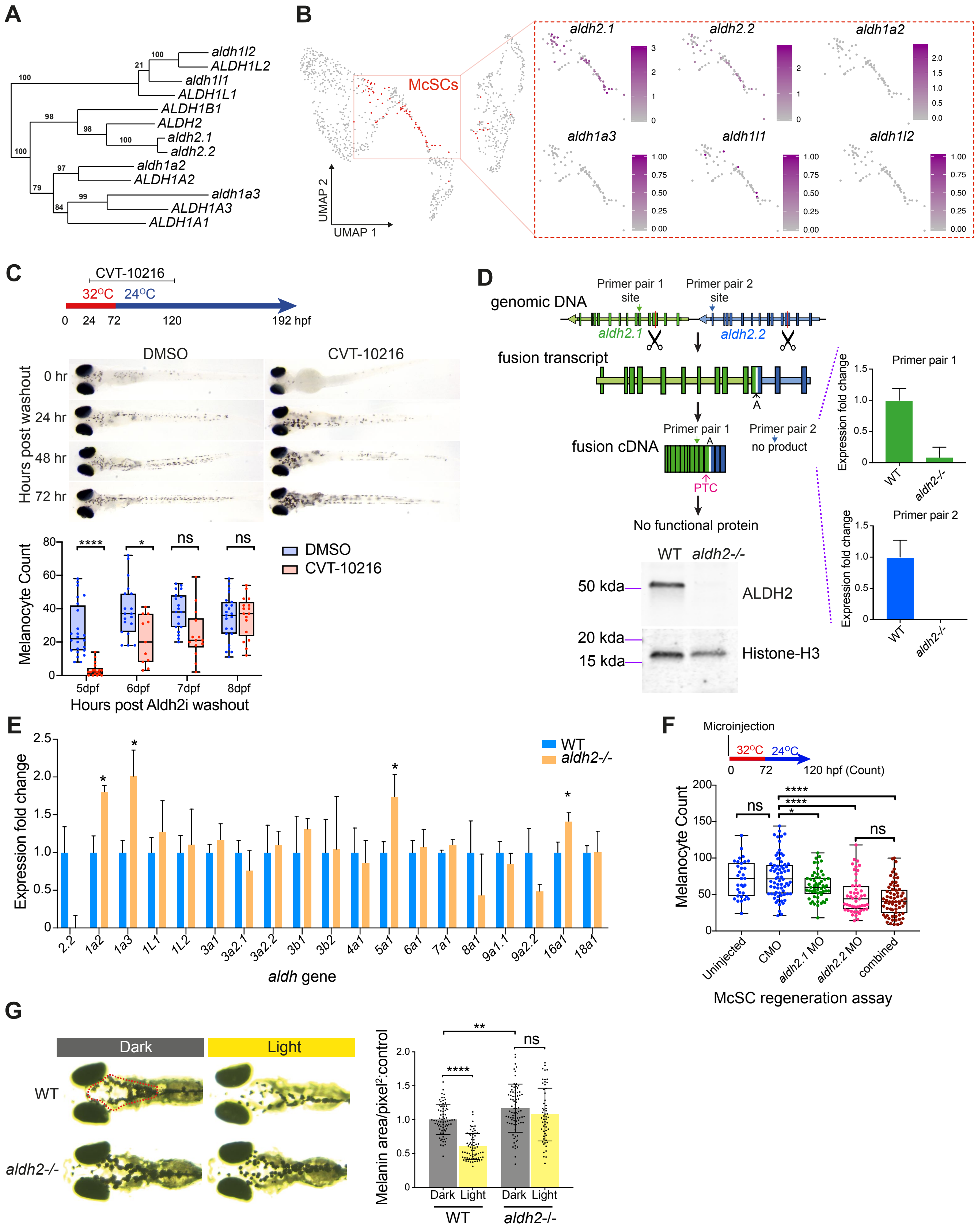

Development • Supplementary information

Fig. S1. *aldh gene* expression in the McSC lineage and Aldh2 loss of activity assays

A. Phylogenetic tree showing the relationship between human *ALDH* and zebrafish *aldh* genes from the ALDH1 and ALDH2 families.

B. As **Fig. 1A**, but showing expression levels of *aldh1* family genes.

C. Extended regeneration assay on *mitfa*^{vc7} embryos following CVT-10216 treatment between 24-120hpf. After washout, larvae were imaged and melanocytes quantified each day. Representative images shown. Each datapoint represents a single larva, 3 experimental replicates. *: p<0.0332, ****: p<0.0001, ns: not significant. Kruskal-Wallis test with Dunn's multiple comparisons.

D. sgRNAs targeting exon 3 of *aldh2.1* and *aldh2.2* were co-injected with Cas9, causing deletion of the intergenic region and creation of a fusion transcript, containing a 1 bp insertion and premature termination codon (PTC). RT-qPCR was performed with primers targeting Primer site 1, persisting in the truncated fusion transcript, and Primer site 2 (excised), see Methods. Housekeeping control: *β-actin*. 3 experimental replicates. Error bars = mean±s.e.m. Western blot analysis of ALDH2 protein in *aldh2-/-* and WT embryos confirms a lack of Aldh2 protein in mutants. Loading control: Histone-H3.

E. RT-qPCR showing *aldh2.2* expression levels relative to other *aldh* genes in 72hpf WT or *aldh2-/-* mutant embryos, normalized to *β-actin*. 3 experimental replicates. Error bars = mean±s.e.m. Asterisks mark *aldh* genes upregulated >1.5-fold in *aldh2-/-* mutants.

F. Regeneration assay on *mitfa*^{vc7} embryos injected with 6 ng of standard control morpholino (CMO), or morpholinos against either/both *aldh2.1* and *aldh2.2*. Regenerated melanocytes are quantified, each datapoint represents a single embryo, 3 experimental replicates. * p<0.0332, **** p<0.0001, ns: not significant. One-way ANOVA with Tukey's multiple comparisons.

G. Representative images shown of embryos after adaptation to dark or light surroundings. Melanin coverage within the red outlined area was quantified. N=3 biological replicates. Error bars = mean±s.d. **: p<0.0021, ****: p<0.0001. One-way ANOVA with Tukey's multiple comparisons.

Development • Supplementary information

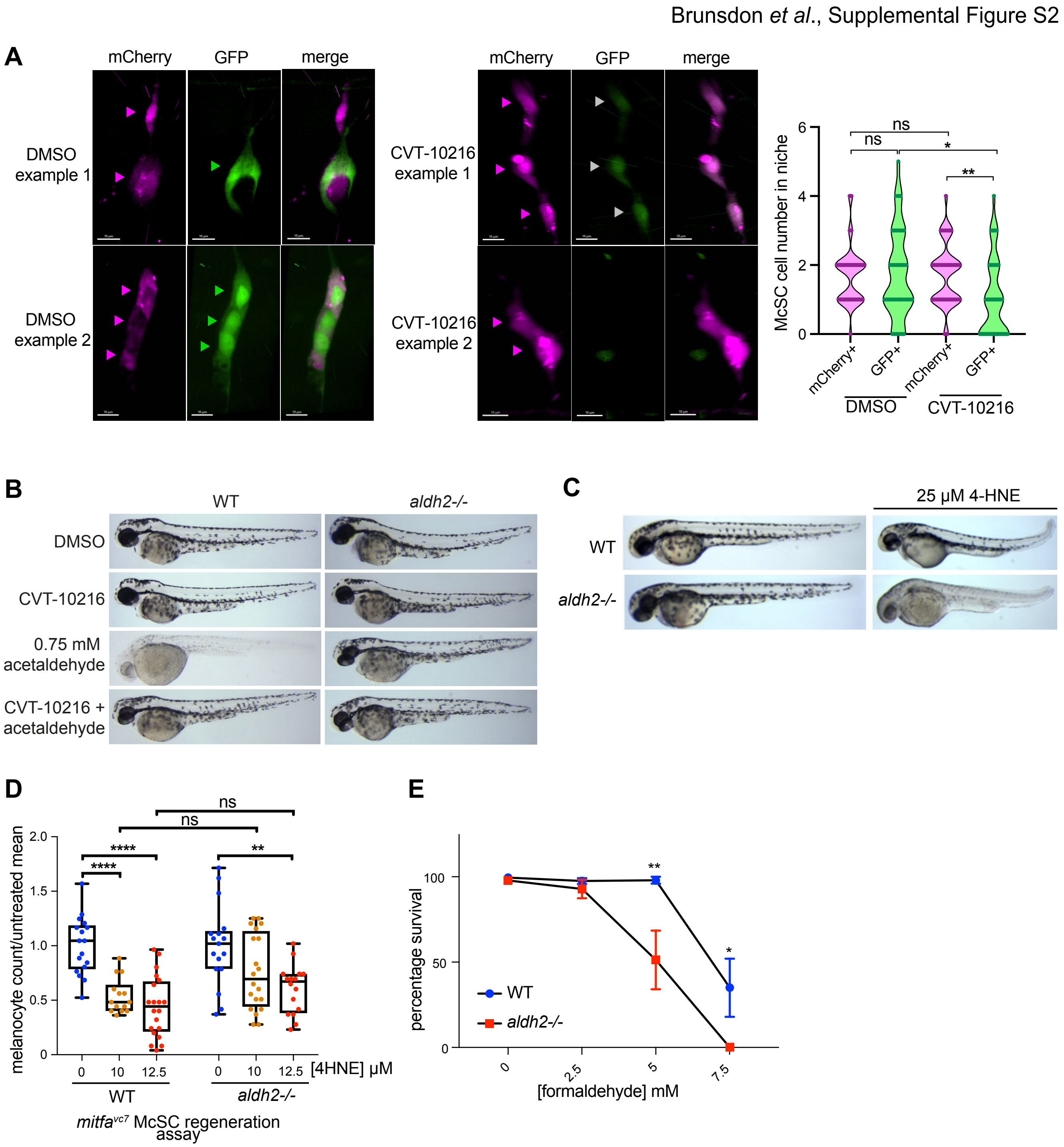

Development • Supplementary information

Fig. S2. Results of aldehyde screen on *aldh2-/-* mutant zebrafish

A. Left: Static images from Movie S1 and S2 (bottom row) plus two additional example McSC niches showing individual *crestin:mCherry* and *mitfa:GFP*-expressing cells (arrowed) during regeneration in embryos treated with either DMSO or CVT-10216, scale bars 10 µm. GFP fluorescence is fainter (gray arrows) or missing after CVT-10216 treatment. Right: Quantification of mCherry+ and GFP+ cells in each niche (3 niches imaged in 12 embryos per condition). Each dot represents a single niche. **: $p<0.0021$, *:$p<0.03$. Kruskall-Wallis test with Dunn's multiple comparisons.

B. Representative images of 72hpf WT and *aldh2-/-* mutant embryos treated with 0.75 mM acetaldehyde with or without CVT-10216. Unexpectedly, Aldh2 loss or deficiency confers resistance to acetaldehyde. 3 experimental replicates, >8 embryos per condition.

C. Representative images of 72hpf WT and *aldh2-/-* mutant embryos treated with 25 µM 4-HNE, showing whole body sensitivity in the mutant. 2 experimental replicates, 15 embryos per condition.

D. $mitfa^{vc7}$ melanocyte regeneration assay and subsequent quantification of embryos treated with increasing doses of 4-HNE, showing no significant difference between controls and *aldh2-/-;* $mitfa^{vc7}$ embryos in terms of reduction in regeneration potential after 4-HNE treatment. For comparison between genotypes, melanocyte numbers were normalized to the average untreated condition for each genotype. 2 experimental replicates, each datapoint represents a single embryo. ** $p<0.0021$, **** $p<0.0001$, ns not significant. One-way ANOVA with Tukey's multiple comparisons.

E. Survival percentage of WT and *aldh2-/-* mutant embryos treated with various concentrations of formaldehyde. 6 experimental replicates, 20 embryos per condition. Error bars = mean±s.e.m. ** $p<0.0021$. Two way ANOVA with Sidak's multiple comparisons.

Development • Supplementary information

Brunsdon *et al.*, Supplemental Figure S3

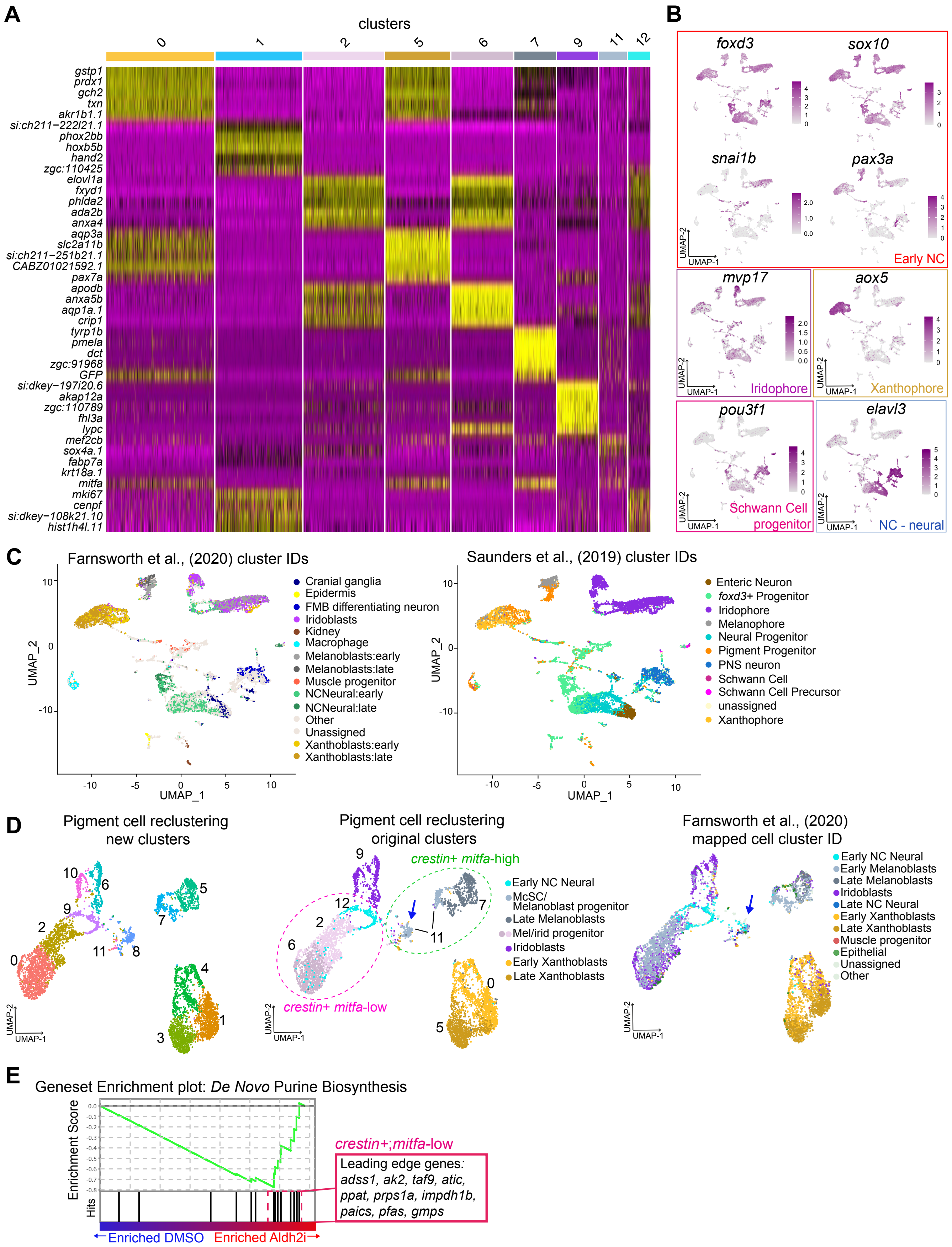

Development • Supplementary information

Fig. S3. Identification of transcriptionally distinct scRNA-seq clusters during McSC regeneration

A. Heatmap showing top 5 cluster-defining genes per selected clusters.

B. UMAP of the combined dataset showing gene expression of early neural crest markers, and non-pigment clusters marked by *pou3f1* (Schwann Cell Progenitors), *mcamb* and *elavl3* marking NC-derived neural cells, *mvp17* marking iridophores, and *aox5* marking xanthophores.

C. UMAP of this scRNA seq data mapped with cell identity annotation from Farnsworth et al (2020) and Saunders et al (2019).

D. Left: UMAP of the combined dataset subsetted by the pigment cells and reclustered into 12 clusters. Center: UMAP annotated with the original clusters, showing the *crestin+;mitfa*-high and *crestin+;mitfa*-low McSCs and split cluster 11. Right: overlap with Farnsworth clusters, showing a portion of original cluster 11 containing a range of neural and pigment cell identities.

E. GSEA enrichment plot of the *de novo* purine biosynthesis signature upregulated in clusters 2,6,12 in CVT-10216 treated embryos compared to control, generated using the Deseq2 output ranked by the Deseq2 test statistic. The green line and y-axis represent the enrichment score of the pathway, that is, the extent of correlation between the DMSO dataset to *de novo* purine synthesis, relative to the Aldh2i dataset. Individual genes within this pathway are represented as vertical black lines, with genes contributing most towards the result - NES -1.18, FDR <25% (Kolganov Smirnov test) - boxed and listed to the right.

Development • Supplementary information

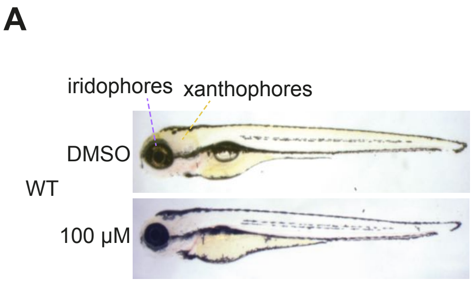

Fig. S4. Validation of Mtx treatment

A. Mtx treated embryos (96 hpf) have lost reflective iridophore pigments (clearly observed in the eye) and yellow xanthophore pigments.

Table S1. scRNA-seq: top 30 cluster markers

Click here to download Table S1

Table S2. scRNA-seq: metrics, clustering information and cell states

Click here to download Table S2

Table S3. Differential expression analysis of *crestin*+ *mitfa-low* vs *crestin*+ *mitfa-high* cells

Click here to download Table S3

Table S4. Differential expression analysis of *crestin+ mitfa-low* cells, DMSO vs CVT-10216

Click here to download Table S4

Table S5. Differential expression analysis of *crestin+ mitfa-high* cells, DMSO vs CVT-10216

Click here to download Table S5

Table S6. Differential expression analysis of iridophore cluster 9, DMSO vs CVT-10216

Click here to download Table S6

Table S7. Oligonucleotide sequences

Click here to download Table S7

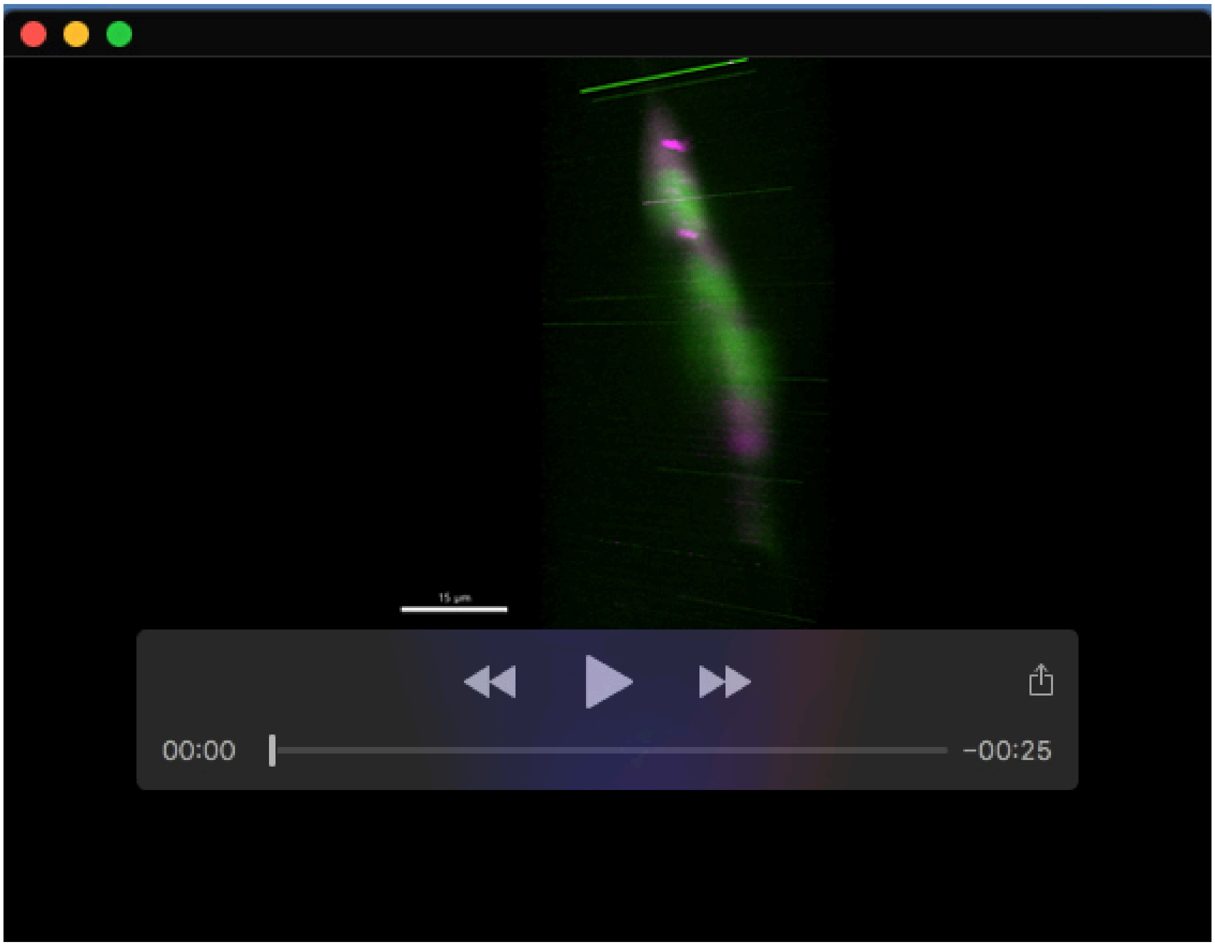

Movie 1. McSCs in a regenerating embryo (DMSO control)

Movie showing 3D rendering of a representative McSC cluster during regeneration. Here, an embryo expressing transgenes *crestin:mCherry* and *mitfa:GFP* embryo is imaged at the onset of regeneration following MoTP treatment. In this example, three cells in the McSC niche express both *GFP* and *mCherry*. See **Fig. S2**, DMSO example 2

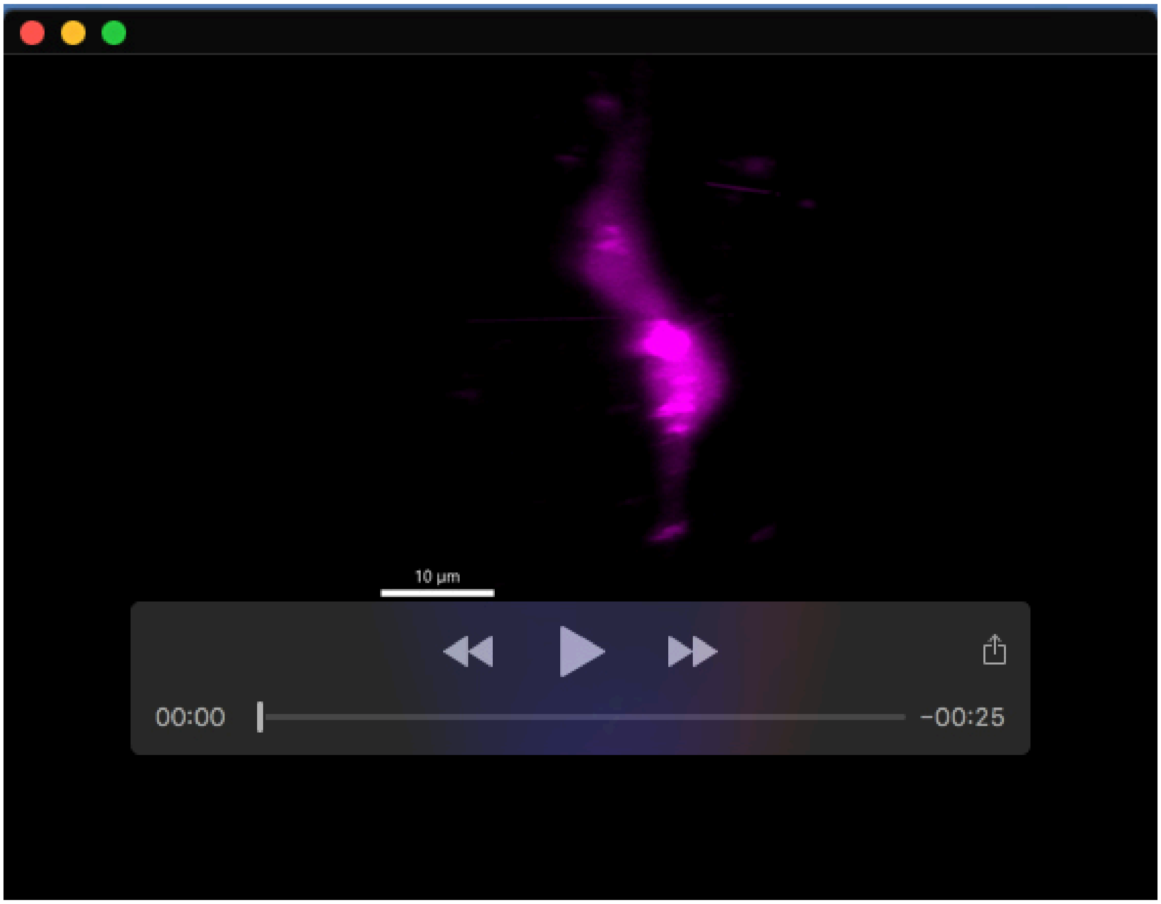

Movie 2. McSCs in a regenerating embryo following ALDH2 inhibitor (CVT-10216) treatment.

Movie showing 3D rendering of a representative McSC cluster during regeneration. Here, an embryo expressing transgenes *crestin:mCherry* and *mitfa:GFP* embryo is imaged at the onset of regeneration during CVT-10216 treatment and following MoTP treatment. In this example, two cells express *mCherry*, but while GFP is detected in a neighbouring cell, it is not expressed in this McSC niche. See **Fig. S2**, CVT-10216 example 2.

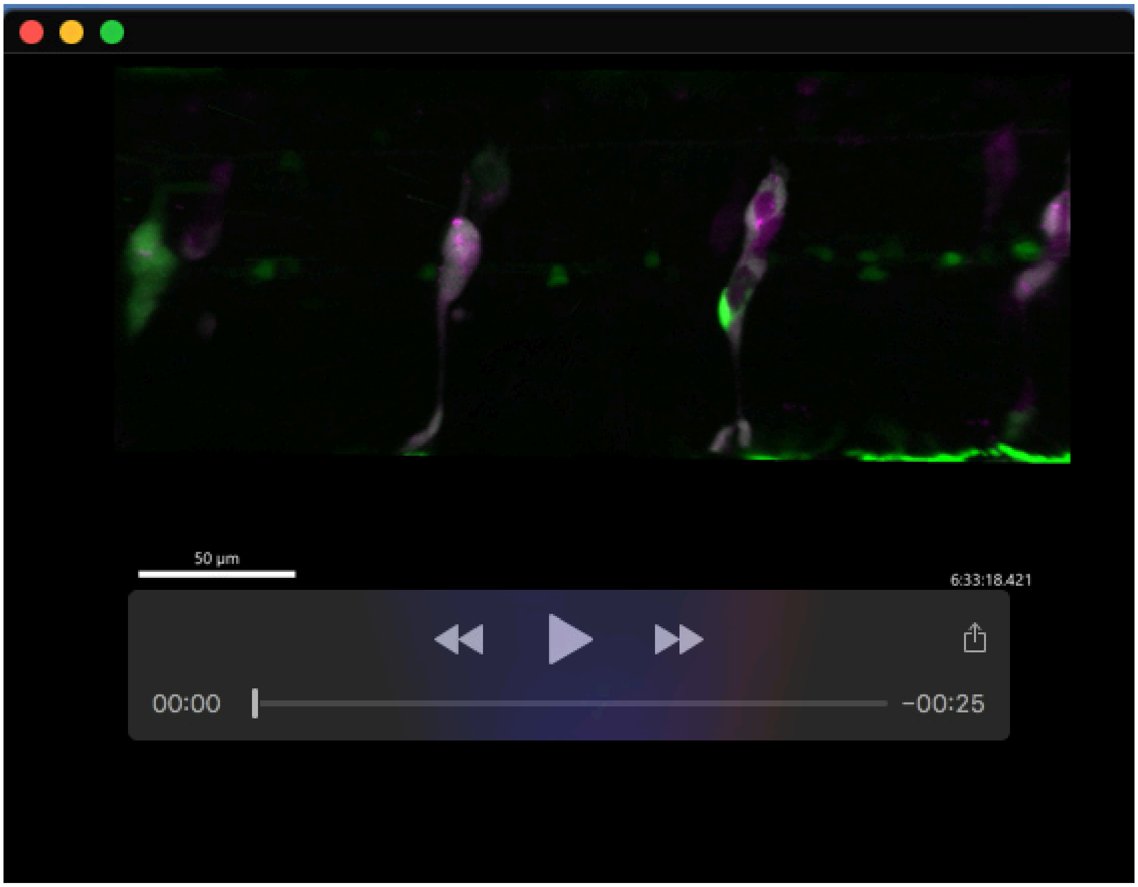

Movie 3. McSCs generate progeny

Time-lapse video of a *Tg(crestin:mCherry;mitfa:GFP)* embryo during McSC regeneration (DMSO control). Embryos were treated with MoTP to kill differentiated melanocytes and initiate melanocyte regeneration. McSCs were followed for over 14 hours (post MoTP washout). McSCs are *crestin+ mitfa-low*, but then during or shortly after cell division, a cell strongly expresses GFP+ to become a *mitfa-high* cell, which then leaves the McSC compartment and migrates towards the epidermis.

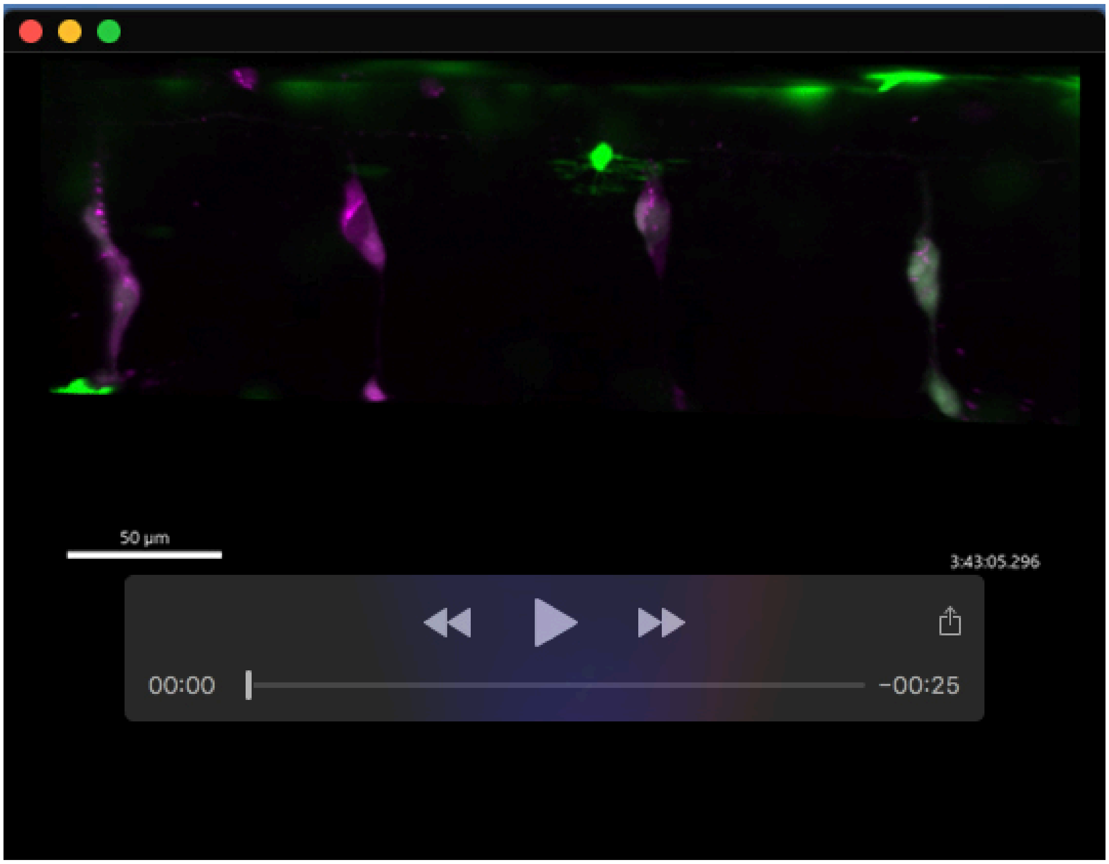

Movie 4. Aldh2i inhibits McSCs ability to generate progeny

Time-lapse video of a *Tg(crestin:mCherry;mitfa:GFP)* embryo during McSC regeneration in the presence of ALDH2i. Embryos were treated with CVT-10216 to inhibit Aldh2, and co-treated with MoTP to kill differentiated melanocytes, and initiate melanocyte regeneration. McSCs were followed for over 14 hours (post MoTP washout, but in the presence of ALDH2i).

Development • Supplementary information
