# Supplementary material for: Aldh2 is a lineage-specific metabolic gatekeeper in melanocyte stem cells
Source: Development. 2022 May 19;149(10):dev200277. doi: 10.1242/dev.200277 (PMC9188749; doi:10.1242/dev.200277)
Supplement: Reviewer comments [file develop-149-200277_review_history.pdf]

Aldh2 is a lineage-specific metabolic gatekeeper in melanocyte stem cellss

Hannah Brunsdon, Alessandro Brombin, Samuel Peterson, John H. Postlethwait and E. Elizabeth Patton
DOI: 10.1242/dev.200277

Editor: Steve Wilson

Review timeline

Original submission:	19 October 2021
Editorial decision:	6 December 2021
First revision received:	7 March 2022
Accepted:	20 April 2022

Original submission

First decision letter

MS ID#: DEVELOP/2021/200277

MS TITLE: Aldh2 is a lineage-specific metabolic gatekeeper in melanocyte stem cells

AUTHORS: Hannah Brunsdon, Alessandro Brombin, Samuel Peterson, John H Postlethwait, and E. Elizabeth Patton

I have now received all the referees' reports on the above manuscript, and have reached a decision. The referees' comments are appended below, or you can access them online: please go to BenchPress and click on the 'Manuscripts with Decisions' queue in the Author Area.

As you will see, the referees like your work, but have somerecommendationsto improve the manuscript, mostly relating to clarificationsratherthan substantial changes/new experiments. After addressing these comments, I will be happy receive a revised version of the manuscript. Please also note that Development will normally permit only one round of major revision.

Please attend to all of the reviewers' comments and ensure that you clearly highlight all changes made in the revised manuscript. Please avoid using 'Tracked changes' in Word files as these are lost in PDF conversion. I should be grateful if you would also provide a point-by-point response detailing how you have dealt with the points raised by the reviewers in the 'Response to Reviewers' box. If you do not agree with any of their criticisms or suggestions please explain clearly why this is so.

Reviewer 1

Advance summary and potential significance to field

The authors explore the role for ALDH in the recruitment of melanocyte stem cells in zebrafish larvae using in vivo imaging with appropriate reporter lines in mutants and drug-treated wild-types, and sc-RNA sequencing . Add-back studies help establish that ALDH is required not to prevent aldehyde mediated toxicity in melanocytes, although it has that function, but to provide an essential substrate for purine synthesis. Analysis of the datasets permits the identification of a quiescent McSC population that activates a neural crest transcriptional program followed by a

© 2022. Published by The Company of Biologists under the terms of the Creative Commons Attribution License (https://creativecommons.org/licenses/by/4.0/).

transition to a proliferative state. The mechanism has been attributed, in part, to ALDH2 activity and its ability to metabolize formaldehyde to formate. The authors unexpectedly find that in the absence of ALDH2, purines rather than pyrimidines are necessary to activate McSCs and generate progeny. The manuscript is well written and experimentally sound. The authors have made publicly available their raw sequencing data which will be well received by the community.

Comments for the author

I have only minor comments:
(1) Figure 1A: Add the developmental stage to the figure and/or figure legend.
(2) Figure 1A: Additional explanation of how the McSC population was classified is necessary. I'm not entirely convinced the red cells are McSCs.
(3) Line 72 and Figure 1C: In the presence of CVT-10216 melanocyte regeneration from McSCs was significantly delayed, have the authors looked beyond 120 hpf? Given the delayed phenotype what stage do melanocytes recover.
(4) Figure 4A: Why was the crestin-mcherry reporter necessary for the single cell analysis?
(5) Line 215: the reference to Fig 5a does not make sense.
(6) Line 287: Typo "that that"
(7) Line 376: What region was quantified using image J? is it the region outline in the DMSO-treated embryo shown in Fig 1C? This should be made explicit. What are the landmarks that define it? Was epinephrine used to make overlapping melanophores easier to distinquish?
(8) The abbreviation of sc should be defined as single cell the first time it is used
(9) Line 157 "To this end, we designed a scRNA-seq analysis of a MoTP melanocyte regeneration experiment in which double transgenic mitfa:GFP; crestin:mCherry embryos were treated with DMSO or CVT-10216 (Fig. 4a). We identified 24 clusters of transcriptionally distinct cell populations by comparing the top 30 variably expressed genes,…" What cells were collected by FACs - both GFP positive and mCherry positive cells or solely double positive cells? The methods section mention gating on mCherry and mGFP but it is not specificed whether single positive or solely double positive cells were collected
(10) Dooley et al 2013 claim to have seen mitfa:gfp-positive cells throughout early larval development.
This paper and Brombin et al report that mitfa:gfp are no longer visible after 120 hpf. These authors are not obliged to explain another group's results but perhaps there is an explanation for the discrepancy?

<u>Reviewer 2</u>

Advance summary and potential significance to field

In this manuscript, Brunsdon et al. define the role of Aldh2-dependent formaldehyde metabolism in melanocyte stem cell (McSC) regeneration in zebrafish.
From scRNAseq data, the authors identify Aldh2 as significantly expressed in McSC's. By using an Aldh2 inhibitor during a melanocyte regeneration assay, they find that Aldh2 is required for McSC regeneration into melanoblasts. Through live fluorescent imaging of the McSC niche in transgenic fish lines, they find that while crestin (representing neural crest identity) is expressed in McSC's upon regeneration initiation, Aldh2 is required to promote mitfa expression in McSC's as well as progeny formation and migration. They then screen various aldehydes as substrates for Aldh2, identifying formaldehyde as the main substrate involved in McSC regeneration. As formate supplementation rescues Aldh2-/- regeneration defects, the authors find that metabolism of formaldehyde to formate is important in regeneration. Next, they hypothesize through scRNAseq analysis that McSC's upregulate 1C metabolism to promote formation of melanoblasts, which they confirm as 1C inhibition causes regeneration defects. Testing the effects of 1C cycle products, they find that purines alone rescue regeneration defects. Taken together, this data suggests that McSC regeneration in zebrafish requires Aldh2-mediated metabolism of formaldehyde to formate, which then enters the 1C cycle to produce purines. This paper would make a significant impact in the field, as not much is known about the role of metabolic products on stem cell regeneration. With minor revisions this manuscript will be of benefit to the readers of Development.

© 2022. Published by The Company of Biologists under the terms of the Creative Commons Attribution License (https://creativecommons.org/licenses/by/4.0/).

Comments for the author

Major Comments:

- In order to study the effects of Aldh2 on regeneration, the authors used mitfa:GFP as a marker of McSC's in the niche. Using this marker, they showed in Figure 2 that Aldh2 inhibition caused a decrease in GFP expression and fewer migratory "progeny" from the niche. Mitfa expression is not unique to McSC's, as it is expressed at various stages of melanocyte differentiation. In referencing Figure 2b, the authors mention that GFP intensity across different McSC clusters was heterogeneous, which could be due to mitfa:GFP marking more differentiated melanocytes. To be more confident that these mitfa:GFP cells seen at the DRG are true McSC's, it would be beneficial to elaborate on markers of McSC identity mentioned in scRNAseq analysis in Figure 4d. In this figure, the authors mention that they saw a "mix of markers consistent with McSC identity" in mitfa:low clusters (clusters 2, 6, 12). Listing a few of these markers and their abundance would provide evidence that this mitfa:GFP population is indeed composed of McSC's.
- The authors show that melanocyte regeneration is affected by Aldh2 activity and 1C metabolism by counting (by eye) pigmented melanocytes that develop. In Figure 2, they also demonstrate, through fluorescence microscopy, the regeneration process of mitfa:GFP McSC's in the DRG niche. However, it would be useful to link these two observations by showing specifically that these mitfa:GFP McSC's truly give rise to the differentiated melanocytes that the authors see by eye. This could be done by imaging distinct mitfa:GFP niches (noting specific region being imaged) daily, accompanied with daily tracking of pigmented melanocyte appearance in that same region. This would strengthen their assumption that the behavior of these GFP+ McSC's they are imaging are truly relevant to the end phenotype.

Minor Comments:

- In Figure 1 when introducing the temperature-sensitive mitfavc7 regeneration assay, it would be beneficial to briefly explain the timeline of melanocyte differentiation, mentioning that embryonic melanocytes develop directly from the neural crest prior to 3 dpf, after which McSC's are called upon for regeneration. This would aid the reader in understanding the time points chosen to study regeneration in this assay.
- Throughout the paper, experiments were performed using two different regeneration assays, mitfavc7 and MoTP, interchangeably. Because the mechanisms of these assays are different, there is a possibility that the acquired data may not be applicable to McSC regeneration widely but rather specific to the given assay.
It would be beneficial to acknowledge this caveat in the conclusion of the paper.
- The authors claim that the melanocyte regeneration results in the Aldh2-/- model prove that Aldh2 inhibitor CVT-10216 is specific for Aldh2, as both methods yielded similar results. However, Figures 1c and 1d indicate that there is slightly less regeneration defect in the Aldh2-/- model compared to the CVT-10216 model, suggesting that CVT-10216 may inhibit other aldehydes. As the authors used CVT-10216 in Figures 2-6, it would be beneficial to mention in the conclusion the caveat of using CVT-10216 over the likely more specific Aldh2-/- model.
- In Figure 2, the authors mention that the decrease in mitfa:GFP expression upon Aldh2 inhibition may be due to a decrease in McSC number or a decrease in mitfa expression. To help parse this out, it would be beneficial to manually outline separate cells in the microscopy images. As is, it is difficult to identify how many cells are present in each niche. If outlining separate cells in these images is not possible, the authors may be able to perform an H&E stain/immunohistochemistry on niche regions to determine cell number.
- In Fig 5e, no representative image is included for the quantitation of 100 uM Mtx.

<u>Reviewer 3</u>

Advance summary and potential significance to field

This manuscript from the Patton lab describes experimental investigation of the role for Aldh2 in melanocyte stem cells (McSCs). The authors show that melanocyte regeneration results from quiescent McSCs in the DRGs switching to a more neural crest (NC)-like identity, and then expressing Aldh2 triggering a metabolic switch to generate progeny pigment cells. The authors have developed a nice assay for identifying the McSCs in living embryos and for imaging their activation, and its inhibition by Aldh2 inhibition/mutation (Fig. 2). They also use innovative substrate and

© 2022. Published by The Company of Biologists under the terms of the Creative Commons Attribution License (https://creativecommons.org/licenses/by/4.0/).

rescue assays to identify the role and mechanism for Aldh2 in McSCs (Fig. 3). The identification of these has implications for humans with defective ALDH2. The work also identifies a novel metabolic control of melanocyte stem cell activity, contributing to identification of a signature perhaps shared across stem cell types.

Comments for the author

Whilst the paper generally shows the careful presentation and clarity characteristic of this lab, I found that I struggled in some sections (especially around Fig. 4), principally due to the sheer complexity of the experiments performed (combining regeneration assay, multiple transgenic labels regeneration inhibitor treatment and rescue)! Summary Fig at end of Fig 6 is excellent.
My key comments are as follows:
Line (l) 75. Melanocyte regeneration is described as 'delayed', but authors do not show that there is further recovery with time, leaving open the question of whether it is 'delayed' or 'partially inhibited'?
l.118 Assertion that McSCs re-express a NC identity seems to be based upon 1 marker, the crestin transgenic reporter. The authors should assess other markers, or tone down claim slightly.
L158 onwards. Experimental rationale for this scRNA-seq experiment and its interpretation needs to be clearer. This whole section is v dense and thus made unnecessarily obscure to the reader. As I understand it, the crestin transgene will be labelling most/all NC derivatives, whereas the mitfa:GFP will label only the melanocytes, including regenerating ones, plus the activated McSCs from which regenerated melanocytes derive. Embryos were treated with either carrier (DMSO; control) or the Aldh2 inhibitor (in which the regeneration process is blocked downstream of activation of McSCs). But later, text refers to 'regenerating pigment clusters inc. melanoblasts'. It needs to be stated explicitly which are these? And do these include xanthophore and iridophore lineages (seem to be high in Aldh2)? If so, why are these regenerating after MoTP treatment? Indeed, what is evidence they are regenerating, rather than simply being labelled by crestin transgene. I think it would help if authors could add new panels to indicate which cell clusters were single and double labelled for transgenes….as it is, text seems to imply that they have been deduced indirectly from expression patterns (l. 175-180). Why is that necessary?
L184+ Needs to stated explicitly whether the higher proportion of crestin+/mitf-high cells is directly assessed, or simply deduced from size of clusters assigned (in previous paragraph) to these populations. It is currently unclear what is observation and what is inference.
L198 Fig. S3d needs explanation for non-expert
Fig. 1b Arrows indicating McSCs with low GFP not visible Fig. 4. How many cell profiles (post-QC) form the dataset for the clustering analysis? (This buried in M&M but would be good to know here)
Fig. 5c States that single channel images are shown alongside merges, but all look merged to me. Consequently hard to assess claim that crestin+mitfa-low cells unchanged by formate treatment.

Minor points:
In general, I found the figure legends to be rather minimal and in some cases figures take a lot of studying to decipher. This could be improved by explicit explanation of all features shown. In some cases the deficiencies were more persistent:
Supp. Fig. 1 Generation of sldh2-/-. Authors need to clarify location of both primers in Primer pairs 1 and 2. How do these relate to the small blue and green arrows (if at all)?
Supp. Fig. 3d. This needs a brief explanation for those unfamiliar with the approach. What is plotted on x axis?

First revision

Author response to reviewers' comments

We are grateful to the Reviewers and Editor for their time and expertise in reviewing our manuscript and that they consider our work *will make a significant impact in the field* and *has implications for humans with defective ALDH2.*

© 2022. Published by The Company of Biologists under the terms of the Creative Commons Attribution License (https://creativecommons.org/licenses/by/4.0/).

Here, we address all comments below and include new experimental data that show:

1. Quantitative analysis of a reversible delay of melanocyte regeneration following Aldh2 inhibition (new **Figure S1C**).
2. Additional analysis of the scRNA-seq datasets for better resolution of McSC identity (**new Figures S1, 4D, and S3D)**
3. Western blotting that shows loss of Aldh2 protein in *aldh2-/-* mutants compared with controls (new **Figure S1D)**.
4. High resolution imaging that shows the number of cells at the niche (new **Figure S2**; new **Movies 1 and 2**).
5. Comparative imaging parameters that may explain some differences in *mitfa:GFP* expression between our work and Dooley et al., 2013 (**Reviewer Figure 1**).

Once again, we thank the Reviewers for their constructive comments, and we very much hope our manuscript is now ready for publication in *Development*.

Reviewer 1

The authors explore the role for ALDH in the recruitment of melanocyte stem cells in zebrafish larvae, using in vivo imaging with appropriate reporter lines in mutants and drug-treated wild-types, and sc-RNA sequencing. Add-back studies help establish that ALDH is required not to prevent aldehyde mediated toxicity in melanocytes, although it has that function, but to provide an essential substrate for purine synthesis. Analysis of the datasets permits the identification of a quiescent McSC population that activates a neural crest transcriptional program followed by a transition to a proliferative state. The mechanism has been attributed, in part, to ALDH2 activity and its ability to metabolize formaldehyde to formate. The authors unexpectedly find that in the absence of ALDH2, purines rather than pyrimidines are necessary to activate McSCs and generate progeny. The manuscript is well written and experimentally sound. The authors have made publicly available their raw sequencing data which will be well received by the community.

We are grateful to the reviewer for their careful review of our study and for indicating that our data will be "well received by the community". With permission from the Editor, we are pleased that our work is already benefitting others through shared access to our raw data.

I have only minor comments:

1. Figure 1A: Add the developmental stage to the figure and/or figure legend.

   Thank you. This has been added to both the Figure and Legend.

2. Figure 1A: Additional explanation of how the McSC population was classified is necessary. I'm not entirely convinced the red cells are McSCs.

   Thank you for this comment. The data for **Figure 1A** was made using scRNA-seq data from Brombin et al., Cell Reports 2022. Briefly, the red cells were found to be a distinct cell population marked transiently by *tfap2b* expression. Through fate mapping and lineage tracing with a *tfap2b* reporter line, they were shown to give rise to all three zebrafish pigment cell types and nerve-associated cells in the adult pattern. Moreover, this *tfap2b:GFP+* population was Erbb dependent, a known trait of McSCs. Additional evidence that McSCs at the niche are stem cells are described by Dooley et al., Development 2013 and Singh et al., Developmental Cell 2016.

   To clarify this in the **Main Text**, we have added the following:

   Recently, we identified an ErbB-dependent developmental tfap2b+ McSC population that we found to be distinct within neural crest and pigment cell lineages, and which lineage tracing analysis showed gave rise to all three zebrafish pigment cell types, including melanocytes, and nerve-associated cells (Brombin et al., 2022).

3. Line 72 and Figure 1C: In the presence of CVT-10216 melanocyte regeneration from McSCs was significantly delayed, have the authors looked beyond 120 hpf? Given the delayed phenotype what stage do melanocytes recover.

© 2022. Published by The Company of Biologists under the terms of the Creative Commons Attribution License (https://creativecommons.org/licenses/by/4.0/).

Thank you for this comment. Upon re-reading our manuscript we appreciate this was not clear because we had placed this data in **Figure S4**. To address this comment and a similar comment below, we have repeated this extended *mitfa^vc7^* regeneration assay to obtain quantitative data (we previously only showed representative images) and moved these data to **Figure S1**. Our data shows that by 72 hours post washout – 8dpf – melanocyte regeneration in the *mitfa^vc7^* had recovered in Aldh2i-treated embryos. We hope it is now easier for the reader to appreciate that Aldh2 inhibition causes a reversible delay in melanocyte regeneration.

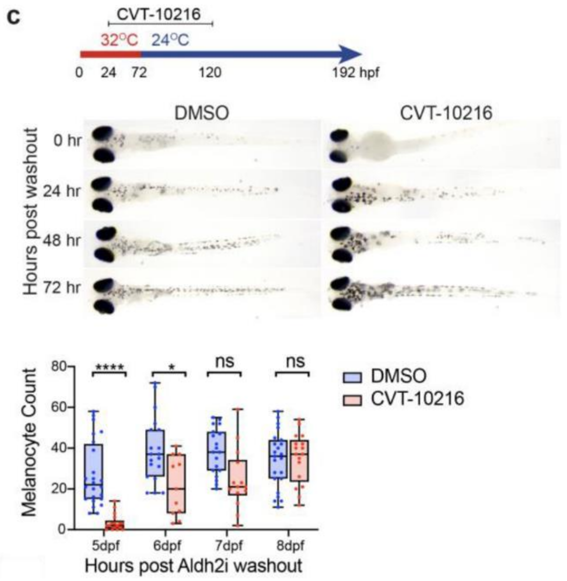

New Figure S1C: Extended regeneration assay on *mitfa^vc7^* embryos treated with CVT-10216 from 24-120hpf. After washout, larvae were imaged, and melanocytes quantified at over time to monitor recovery/continuation of melanocyte regeneration. Representative images are shown from >5 embryos per condition, 3 biological replicates. *: p<0.0332, ****: p<0.0001, ns: not significant. Kruskal-Wallis test with Dunn's multiple comparisons.

4. Figure 4A: Why was the crestin-mCherry reporter necessary for the single cell analysis?

   We included *crestin:Cherry* in the scRNA-seq analysis because we wanted to study the McSC during the Aldh2-dependent metabolic transition identified in **Figure 2**. In **Figure 2 B-D** we show that the McSCs express both *crestin:mCherry* and *mitfa:GFP*, but that the ALDH2i delays the ability of *crestin:Cherry*+ McSCs to generate *mitfa:GFP* progeny. Thus, it was necessary to include the transgenic *crestin:mCherry* reporter line in the design of the experiment.

5. Line 215: the reference to Fig 5a does not make sense.

   We apologise for this and appreciate that the initial schematic reference had become illogical after revisions to the manuscript order. We have reordered Figure 5 and associated references so that the schematic diagram appears later, and hope that this improves the flow and coherence of the text. Thank you for identifying this issue.

6. Line 287: Typo "that that"

   Thank you, this has been amended.

7. Line 376: What region was quantified using image J? is it the region outline in the DMSO-treated embryo shown in Fig 1C? This should be made explicit. What are the landmarks that define it? Was epinephrine used to make overlapping melanophores easier to distinguish?

   Thank you for this comment. We have now outlined the region demarcating the counting area more thickly in **Figure 1C** and have edited the legends and methods to indicate more clearly that this region is uniform across all instances of melanocyte quantification. We did not use epinephrine in

© 2022. Published by The Company of Biologists under the terms of the Creative Commons Attribution License (https://creativecommons.org/licenses/by/4.0/).

this experiment, as regenerated melanocytes of 5dpf embryos imaged under light are punctate enough to distinguish cell boundaries.

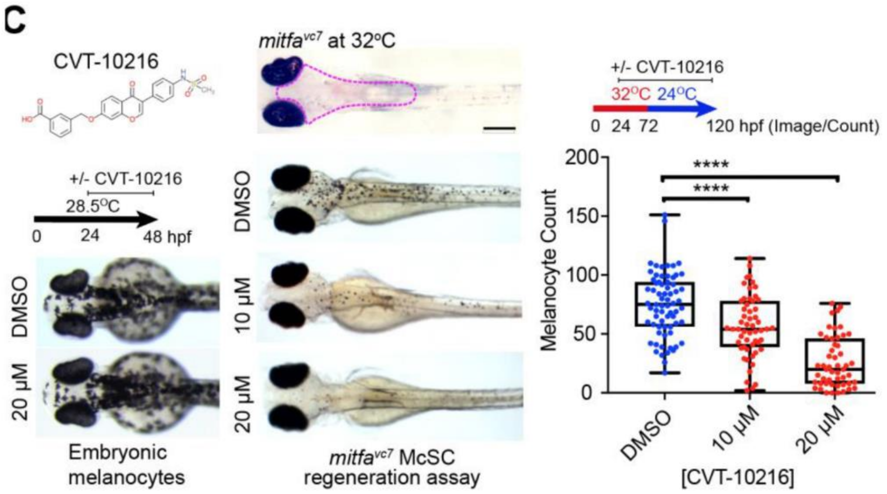

***Figure 1C**: Representative images of wild type embryos treated +/- CVT-10216 during development (embryonic melanocytes) or in an McSC regeneration assay. Regenerated melanocytes were quantified within a consistent region delineated by the magenta dotted line on the non-regenerating control embryo (top). One data point plotted per embryo. Scale bar = 500 µm. **** p<0.0001. One-way ANOVA with Tukey's multiple comparisons test. 4 experimental replicates.*

The **Methods** now reads:

*After fixation, embryos were imaged, and melanocytes counted using the Image J CellCounter plugin within a consistent dorsal area outlined in **Fig 1C**. Embryos were imaged dorsally, and only in-focus dorsal surface melanocytes counted. For the anterior and posterior bounds, anatomical landmarks used include the anterior-most portion of the head but excluding any in-focus melanocytes around the mouth. Posteriorly, we counted until the point at which the yolk 'pinches off' as it meets the tail. This gave a uniform and wide area within which to count melanocytes consistently and gauge differences in number between drug treatments.*

8. The abbreviation of sc should be defined as single cell the first time it is used

Thank you, this has been amended.

9. Line 157 "To this end, we designed a scRNA-seq analysis of a MoTP melanocyte regeneration experiment in which double transgenic *mitfa:GFP*; *crestin:mCherry* embryos were treated with gating on mCherry and mGFP but it is not specified whether single positive or solely double positive cells were collected

Thank you for bringing this to our attention, and we apologise that this was confusing. We have edited the **Main Text** and **Methods** for clarity.

Main Text:

*To this end, we designed a scRNA-seq analysis of a MoTP melanocyte regeneration experiment in which double transgenic mitfa:GFP; crestin:mCherry embryos were treated with DMSO or CVT-10216, and then GFP+, mCherry+ and double+ cells sorted together by FACS and processed for sequencing using the 10x protocol **(Fig. 4A)**.*

Methods:

10,000 GFP+, mCherry+ or double+ cells per treatment condition were sorted together into 100 µl of 0.04% BSA/PBS and processed for the 10x protocol.

© 2022. Published by The Company of Biologists under the terms of the Creative Commons Attribution License (https://creativecommons.org/licenses/by/4.0/).

10. Dooley et al 2013 claim to have seen mitfa:gfp-positive cells throughout early larval development. This paper and Brombin et al report that mitfa:gfp are no longer visible after 120 hpf. These authors are not obliged to explain another group's results but perhaps there is an explanation for the discrepancy?

Thank you for this insightful comment.

We would like to emphasise that we find Dooley et al., 2013 to be a landmark paper and our findings are generally in excellent agreement with their work.
We both use the same transgene although the differing integration site might explain some differences in reporter transgene expression. However, we think that a more plausible explanation might be how the images were acquired and processed **(Reviewer Figure 1)**.

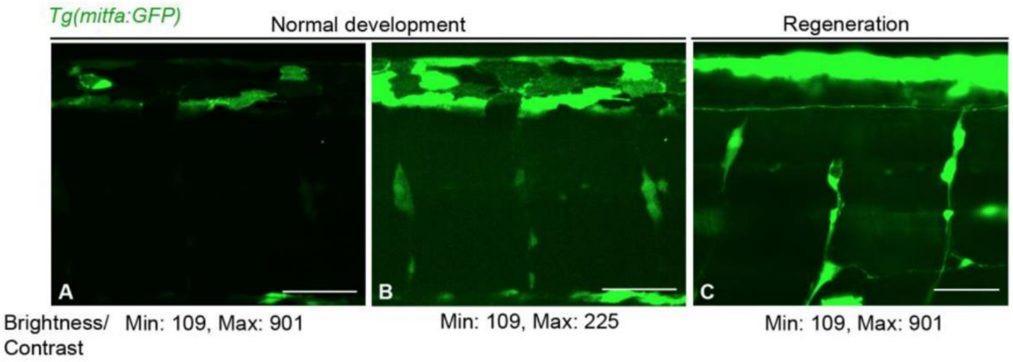

Reviewer Figure 1: Maximum projection Z-stacks of McSC niches during development and regeneration using different brightness/contrast settings.

In **Reviewer Figure 1**, **(A)** is a view of a **non-regenerating** *Tg(mitfa:GFP)* embryo at 96 hpf, a sibling of that shown in **Figure 2C**. These are the imaging conditions we use in this paper. **(B)** is the same, but with the brightness and contrast values altered to give a much more restricted dynamic range to the point where the fluorescence is quite grainy and there is some autofluorescence from xanthophores that is evident. As a point of comparison, *GFP* fluorescence in a **regenerating embryo (C)** is much brighter at the same settings as in normal development. Thus, there is evidently still some GFP expression in non-regenerating embryos, but at a much lower level than in regeneration, and not detectable using the same imaging conditions. Similarly, differences in lasers may account for differences in detection levels.

Alternatively, we typically follow transgenes in hemizygous animals, which could alter detection levels. We have ensured that these breeding strategies are clear in the **Methods**.

To quantify the area of GFP or mCherry-expressing cells within niches, homozygous Tg(mitfa:GFP) fish were outcrossed with non-fluorescent or Tg(crestin:mCherry) fish to obtain embryos with similar levels of transgene expression

While we cannot know exactly why there are differences between these two studies, we (this study), (Brombin et al. 2022), clearly see *mitfa:GFP* fluorescence fading before being reactivated, and it is much lower than in activated McSCs poised to or in the process of regenerating (**Fig. 2C** in this study, Figure 1G in Brombin et al. 2022). We believe these are biologically meaningful differences during regeneration.
To address this issue, we now include the following edits in the **Main Text.**

Alternatively, McSCs may be present but expressing only low ~~(or no)~~ mitfa:GFP under conditions of ALDH2 inhibition.

Although Dooley et al., (2013) detect mitfa:GFP expression at the niche throughout development, we consistently see a down regulation of mitfa:GFP expression in McSCs following establishment at

© 2022. Published by The Company of Biologists under the terms of the Creative Commons Attribution License (https://creativecommons.org/licenses/by/4.0/).

*the niche in non-regenerative conditions (**Figure 2C**; Brombin et al., 2022); these differences may possibly due to differences in imaging parameters and/or transgene expression.*

Reviewer 2

In this manuscript, Brunsdon et al. define the role of Aldh2-dependent formaldehyde metabolism in melanocyte stem cell (McSC) regeneration in zebrafish. From scRNAseq data, the authors identify Aldh2 as significantly expressed in McSCs. By using an Aldh2 inhibitor during a melanocyte regeneration assay, they find that Aldh2 is required for McSC regeneration into melanoblasts. Through live fluorescent imaging of the McSC niche in transgenic fish lines, they find that while crestin (representing neural crest identity) is expressed in McSC's upon regeneration initiation, Aldh2 is required to promote mitfa expression in McSC's as well as progeny formation and migration. They then screen various aldehydes as substrates for Aldh2, identifying formaldehyde as the main substrate involved in McSC regeneration. As formate supplementation rescues Aldh2-/- regeneration defects, the authors find that metabolism of formaldehyde to formate is important in regeneration. Next, they hypothesize through scRNAseq analysis that McSC's upregulate 1C metabolism to promote formation of melanoblasts, which they confirm as 1C inhibition causes regeneration defects. Testing the effects of 1C cycle products, they find that purines alone rescue regeneration defects. Taken together, this data suggests that McSC regeneration in zebrafish requires Aldh2-mediated metabolism of formaldehyde to formate, which then enters the 1C cycle to produce purines. This paper would make a significant impact in the field, as not much is known about the role of metabolic products on stem cell regeneration. With minor revisions this manuscript will be of benefit to the readers of Development.

We are grateful to the Reviewer for these comments, and delighted that they consider our work to be of benefit to the readers of *Development* readers and of "significant impact in the field".

Major Comments:

1. In order to study the effects of Aldh2 on regeneration, the authors used mitfa:GFP as a marker of McSCs in the niche. Using this marker, they showed in Figure 2 that Aldh2 inhibition caused a decrease in GFP expression and fewer migratory “progeny” from the niche. Mitfa expression is not unique to McSCs, as it is expressed at various stages of melanocyte differentiation. In referencing Figure 2b, the authors mention that GFP intensity across different McSC clusters was heterogeneous, which could be due to mitfa:GFP marking more differentiated melanocytes. To be more confident that these mitfa:GFP cells seen at the DRG are true McSCs, it would be beneficial to elaborate on markers of McSC identity mentioned in scRNA-seq analysis in Figure 4d. In this figure, the authors mention that they saw a “mix of markers consistent with McSC identity” in mitfa:low clusters (clusters 2, 6, 12). Listing a few of these markers and their abundance would provide evidence that this mitfa:GFP population is indeed composed of McSCs.

We thank you the Reviewer for these important comments.

We agree that the *mitfa:GFP* transgene is not specific to McSCs, however, we can identify McSCs by their location within the embryo. McSCs were first proposed by Budi et al., Development 2008 and Johnson et al., Dev Biol 2011, and the location of these cells as closely associated with the emerging doral root ganglion (DRG) was demonstrated by the Parichy laboratory (Budi et al., Plos Genetics 2011) and the Nusslein-Volhard laboratory (Dooley et al., Development 2013).

To identify marker genes of these cells we recently performed a scRNA-seq analysis of zebrafish embryos expressing the transgenes *Tg*(*mitfa:GFP crestin:mCherry*) at 24 hpf and found that they transiently express *tfap2b* during McSC establishment at the niche (Brombin et al., Cell Reports 2022). Through lineage tracing we were able to show that these cells give rise to all three pigment cell types in the adult pattern, as well as nerve-associated cells.

Importantly, we find that *tfap2b*+ McSCs express neural crest genes (*sox10*, *crestin*) and the marker genes for all three pigment cell lineages (melanophores, iridophores and xanthophores; MIX) and neural cell types. This was our meaning when we referred to a "mix of markers consistent with McSC identity", as we also see multiple pigment cell and neuronal cell types in cluster 12, for example.

© 2022. Published by The Company of Biologists under the terms of the Creative Commons Attribution License (https://creativecommons.org/licenses/by/4.0/).

However, to address the Reviewer comment further, we have reanalysed our data by sub-setting only the pigment cell lineages (clusters 2, 6, 12 (*crestin+ mitfa-low*), clusters 7 and 11 (*crestin+ mitfa-high*) as well as clusters 0, 5 (xanthophores) and cluster 9 (iridophores)), and then clustered them with Seurat.

This new analysis identified 12 transcriptionally distinct pigment cell clusters (**new Figure S3D**). Interestingly, comparing to the original cluster identity, cluster 11 was divided into two distinct clusters (7, 8). This 'new' cluster 8 – corresponding to a portion of *crestin+ mitfa*-high McSCs – also expresses markers consistent with early NC neural cells, and all three pigment cell types when remapped onto the Farnsworth dataset. This suggests that this population also expresses markers associated with McSCs, possibly capturing the McSCs as they become activated to generate progeny.

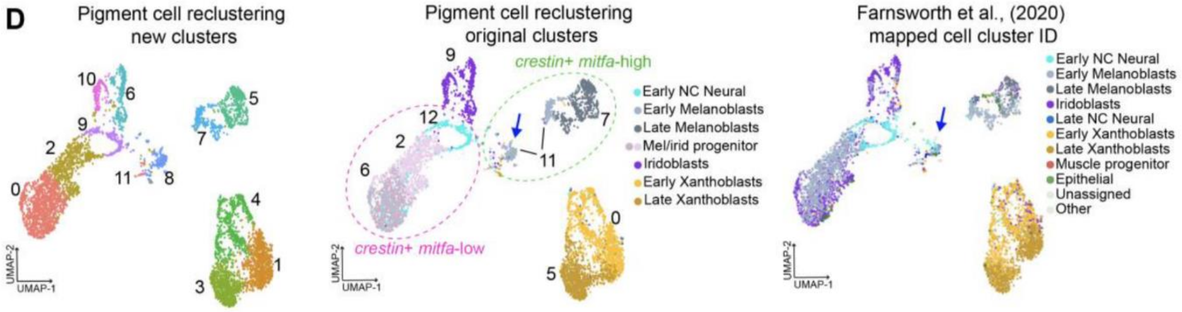

New Figure S3D: *Left: UMAP of the combined dataset subsetted by the pigment cells and reclustered into 12 clusters. Middle: UMAP annotated with the original clusters, showing the crestin+;mitfa-high and crestin+;mitfa-low McSCs and split cluster 11. Right: overlap with Farnsworth clusters, showing a portion of original cluster 11 containing a range of neural and pigment cell identities.*

Therefore, consistent with our imaging analysis and that of others (Dooley et al., 2013; Brombin et al., 2022), we propose that both *crestin+ mitfa-low* and at least some *crestin+ mitfa-high* are McSCs, albeit at different stages in their trajectory.

We appreciate McSC biology is a nascent research area and that the language we use to describe these cells is not yet well characterised. We have therefore amended the **Main text** as follows:
We identified 24 clusters of transcriptionally distinct cell populations by comparing the top 30 variably expressed genes, generating UMAPs featuring expression of known lineage-defining NC genes, and mapping the cluster identities from two recent zebrafish scRNA publications onto our data (Saunders et al. 2019; Farnsworth et al. 2020) ***(Fig. 4B, C; Fig. S3; Tables S1, 2)****.*

*As crestin:mCherry is expressed in a wide range of neural crest-derived cell populations (Kaufman et al. 2016), we captured both pigment cell lineages and cells of the neural lineage. Clusters 7 and 11 expressed crestin and mitfa, with cluster 7 enriched for later stage melanoblast markers such as dct. Cells in clusters 2, 6 and 12 expressed crestin, but low mitfa, and mapping previously published scRNA-seq datasets onto this cluster reveals they contained a mix of pigment and neural cell identity markers, consistent with stem cell identity (Farnsworth et al. 2020; Brombin et al. 2022). Upon closer analyses of pigment cells, we found a subset of cluster 11 also shared these characteristics, suggesting that these are also McSCs (****Fig. S3****). aldh2.2 and aldh2.1 were expressed across multiple pigment cell clusters including McSCs and melanoblasts* ***(Fig. 4C)****.*

2. The authors show that melanocyte regeneration is affected by Aldh2 activity and 1C metabolism by counting (by eye) pigmented melanocytes that develop. In Figure 2, they also demonstrate, through fluorescence microscopy, the regeneration process of mitfa:GFP McSC's in the DRG niche. However, it would be useful to link these two observations by showing specifically that these mitfa:GFP McSC's truly give rise to the differentiated melanocytes that the authors see by eye. This could be done by imaging distinct mitfa:GFP niches (noting specific region being imaged) daily, accompanied with daily tracking of pigmented melanocyte appearance in that same region. This would strengthen their assumption that the behaviour of these GFP+ McSC's they are imaging are truly relevant to the end phenotype.

© 2022. Published by The Company of Biologists under the terms of the Creative Commons Attribution License (https://creativecommons.org/licenses/by/4.0/).

Thank you for this important comment regarding the fate of McSCs. The journey of McSC progeny to its differentiated pigmented melanocyte has already been beautifully shown by Dooley et al., Development 2013, in which they use live imaging to trace McSC progeny migrating along nerves as they become pigmented during regeneration, and show that McSC progeny can regenerate all components of the larval melanocyte pattern. More recently, long term lineage tracing experiments by Singh et al., Dev Cell 2016 using a Cre/loxP-mediated recombination shows tracing of a single *sox10*+ clone at the DRG giving rise to pigmented melanophores as well as other pigment cell types and neurons over a period of 40 days. Supporting the evidence that McSCs give rise to differentiated melanocytes, our own recent work (Brombin et al., Cell Reports 2022), uses lineage tracing of individual *tfap2b*+ McSCs from early development to show that these McSCs at the DRG give rise to all pigment cell types in the adult, including pigmented melanocytes.

We appreciate the comment from the Reviewer, and have now included an additional sentence in the **Introduction** as follows:

In zebrafish, nerve-associated McSCs are an on-demand regenerative population for zebrafish at all stages, and the cell-of-origin for multiple pigment cell types as the zebrafish grows to become an adult (Budi et al. 2008; Budi et al. 2011; Dooley et al. 2013; Singh et al. 2016; Brombin et al. 2022). Imaging analysis over time as well as lineage-tracing studies show McSC progeny directly give rise to pigmented melanocytes (Dooley et al. 2013; Singh et al. 2016; Brombin et al. 2022).
Minor Comments:

3. In Figure 1 when introducing the temperature-sensitive mitfavc7 regeneration assay, it would be beneficial to briefly explain the timeline of melanocyte differentiation, mentioning that embryonic melanocytes develop directly from the neural crest prior to 3 dpf, after which McSC's are called upon for regeneration. This would aid the reader in understanding the time points chosen to study regeneration in this assay.

Thank you for this helpful comment, and we agree this is an important point. We have added the following sentences to the text to clarify the timepoints chosen for regeneration experiment time points.

In wild-type embryos, neural crest-derived embryonic melanocytes pigment the epidermis during the first 72 hours of development before McSCs are activated. The mitfa^{vc7}regeneration model allows us to bypass embryonic pigmentation by growing mitfa^{vc7} embryos at higher temperatures (such that mitfa is spliced incorrectly) to deplete Mitfa activity during this 72 hour period. After this, melanocyte regeneration can be activated from McSCs in mitfa^{vc7} embryos by lowering the water temperature to a level permissive for correct splicing of mitfa, thereby restoring its activity and allowing melanocytes to regenerate from McSCs over a period of 48 hours (Johnson et al. 2011).

4. Throughout the paper, experiments were performed using two different regeneration assays, mitfavc7 and MoTP, interchangeably. Because the mechanisms of these assays are different, there is a possibility that the acquired data may not be applicable to McSC regeneration widely but rather specific to the given assay. It would be beneficial to acknowledge this caveat in the conclusion of the paper.

Thank you, we agree that the two regeneration assays used here are not the same mechanisms. We imagine that as more research is done in this area, different pharmacological and genetic methods will likely provide a more holistic view of McSC regeneration. However, with great respect, we take a slightly different view from the Reviewer, and suggest that finding an Aldh2-dependent requirement in McSCs through two independent regenerations assays is a strength of our work and indicates that are findings are not restricted to one specific regeneration condition.

5. The authors claim that the melanocyte regeneration results in the Aldh2-/- model prove that Aldh2 inhibitor CVT-10216 is specific for Aldh2, as both methods yielded similar results. However, Figures 1c and 1d indicate that there is slightly less regeneration defect in the Aldh2-/- model compared to the CVT-10216 model, suggesting that CVT-10216 may inhibit other aldehydes. As the authors used CVT-10216 in Figures 2-6, it would be beneficial to mention in the conclusion the caveat of using CVT-10216 over the likely more specific Aldh2-/- model.

© 2022. Published by The Company of Biologists under the terms of the Creative Commons Attribution License (https://creativecommons.org/licenses/by/4.0/).

Thank you very much for this comment. Both genetic and chemical approaches have their strengths and weaknesses. Even though we selected CVT-10216 based on its published superior specificity towards Aldh2 over other Aldh enzymes relative to other Aldh2 inhibitors such as Daidzin and Disulfiram, it will undoubtedly also inhibit other Aldh enzymes to an extent, in particular Aldh1a2 (albeit 40x less (Chen et al 2014)). We are hopeful that the impact of this will be minimal in this context because of the relative lack of expression of the most closely related Aldh enzymes in McSCs.

We now include **revised Figure S1** to show *aldh* gene expression in the McSCs dataset from Brombin et al., 2022.

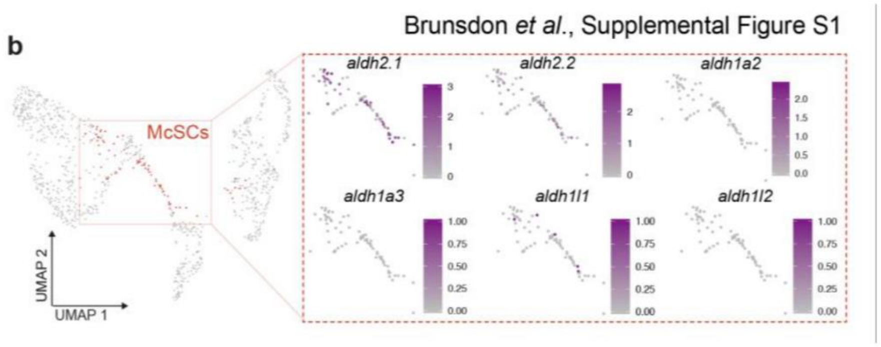

That said, we interpret our results differently, and in this case, believe that CVT-10216 is the better tool for this study over the *aldh2-/-* genetic model.

We suspect that *aldh2-/-* mutants display genetic compensation in this lineage:

1. RT-qPCR showed mutant *ALDH2* cDNA was reduced compared to wild type (**Fig. S1**), suggesting that nonsense mediated decay was degrading the aberrant transcript after detecting the PTC. This is a known trigger for upregulation of closely related genes to compensate, and the proposed reason for discrepancies between morpholino-mediated knockdown and genetic knockout zebrafish phenotypes (El-Brolosy et al. 2019).

2. Following on from above, we observed upregulation of *aldh1a2*, *aldh1a3*, *aldh5a1* and *aldh16a1* by RT-qPCR in *aldh2-/-* mutant embryos compared to wild type (**Fig. S1**).

3. As indicated in the Text, after multiple rounds of breeding the *aldh2-/-* mutant phenotype was lessened. We have now expanded this statement to include the possibility of genetic compensation. "We noticed that after multiple rounds of breeding of our *aldh2-/-* mutants, the melanocyte regeneration phenotype was lessened. This was coupled with transcriptional upregulation of other *aldh* enzyme family members suggesting some plasticity in *aldh* expression in regeneration and the possibility of genetic compensation by other Aldh enzymes (El-Brolosy et al. 2019) **(Fig. S1).**"

4. Translation blocking morpholinos against either or both *aldh2.1* and *aldh2.2* show a reduction in melanocyte regeneration in *mitfa^{vc7}* embryos, similar to the effects of CVT-10216 (**Fig. S1**).

We therefore feel that CVT-10216 is sufficiently selective for Aldh2 as it closely mimics the morpholino experiment, and that it was the best approach to use for the scRNA-seq and formate/purine rescue experiments.

However, the Reviewer is correct to indicate that chemicals can have additional targets, and we have now revised the Discussion as follows:

*We use a combination of aldh2 genetic mutants, morpholino knockdown studies and a highly selective ALDH2 inhibitor to reveal the function for Aldh2 in McSC metabolism (**Figure 1, Figure S1**). Given the selective expression of aldh2 enzymes in the McSC (**Figure S1**) and the high selectivity of CVT-10216 for ALDH2 over other ALDH enzymes (Chen et al. 2014), Aldh2 is likely the primary target of CVT-10216 in the McSC context, although additional studies will be required to understand if other ALDH enzymes are targets of CVT-10216 in vivo.*

© 2022. Published by The Company of Biologists under the terms of the Creative Commons Attribution License (https://creativecommons.org/licenses/by/4.0/).

6. In Figure 2, the authors mention that the decrease in mitfa:GFP expression upon Aldh2 inhibition may be due to a decrease in McSC number or a decrease in mitfa expression. To help parse this out, it would be beneficial to manually outline separate cells in the microscopy images. As is, it is difficult to identify how many cells are present in each niche. If outlining separate cells in these images is not possible, the authors may be able to perform an H&E stain/immunohistochemistry on niche regions to determine cell number.

   Thank you for this suggestion. We agree that that it would be useful to know how many cells are usually within each McSC niche, as this hasn't been measured directly before, and how the number of *mitfa:GFP* cells will be affected by Aldh2 inhibition.

   Unfortunately, we experienced technical difficulties attempting the immuno-staining approach on fixed embryos.

   However, we did have more success by imaging niches at higher magnification and with thinner optical sections, enabling us to closely visualise individual cells in 3D using Imaris software. We have added these data to **Fig. S2**, **Movie S1, S2.** We find that there is no difference between the number of *crestin:mCherry*-expressing cells at the niche, however we do find a strong reduction in both the number of *mitfa:GFP* expressing cells, and the expression of GFP itself. This further supports our model that Aldh2 activity is required for activated *crestin:mCherry* McSCs to generate *mitfa:GFP* progeny.

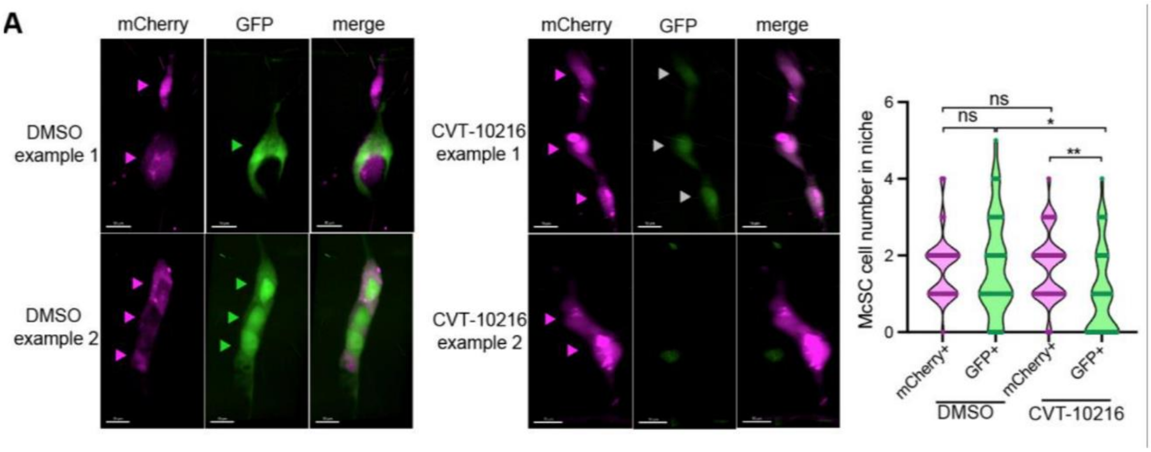

New Figure S2: *Left: Static images from Movie S1 and S2 (bottom row) plus two additional example McSC niches showing individual crestin:mCherry and mitfa:GFP cells (arrowed) during regeneration in embryos treated with either DMSO or CVT-10216. GFP fluorescence is fainter (gray arrows) or missing after CVT-10216 treatment. Right: Quantification of mCherry+ and GFP+ cells in each niche (3 niches imaged in 12 embryos per condition). Each dot represents a single niche. **: p<0.0021, *:p<0.03. Kruskall-Wallis test with* Dunn's multiple comparisons.

   We have now included new **Figure S2**, and **Movies S1 and S2**, and modified the text as follows:

   *Upon ALDH2i treatment, and as seen in **Fig. 2A**, we again observed a specific and strong reduction of GFP in McSCs, with mCherry+ McSCs being still clearly visible. Imaging niches at a higher magnification revealed a significant reduction in mitfa:GFP cells within McSC niches (**Fig. 2B**; **Fig.S2**; **Movie S1**, **S2**).*

7. In Fig 5e, no representative image is included for the quantitation of 100 uM Mtx.

   Thank you for spotting this, **Figure 5** has now been amended.

© 2022. Published by The Company of Biologists under the terms of the Creative Commons Attribution License (https://creativecommons.org/licenses/by/4.0/).

Reviewer 3

This manuscript from the Patton lab describes experimental investigation of the role for Aldh2 in melanocyte stem cells (McSCs). The authors show that melanocyte regeneration results from quiescent McSCs in the DRGs switching to a more neural crest (NC)-like identity, and then expressing Aldh2 triggering a metabolic switch to generate progeny pigment cells. The authors have developed a nice assay for identifying the McSCs in living embryos and for imaging their activation, and its inhibition by Aldh2 inhibition/mutation (Fig. 2). They also use innovative substrate and rescue assays to identify the role and mechanism for Aldh2 in McSCs (Fig. 3). The identification of these has implications for humans with defective ALDH2. The work also identifies a novel metabolic control of melanocyte stem cell activity, contributing to identification of a signature perhaps shared across stem cell types.

Reviewer 3 Comments for the Author...

Whilst the paper generally shows the careful presentation and clarity characteristic of this lab, I found that I struggled in some sections (especially around Fig. 4), principally due to the sheer complexity of the experiments performed (combining regeneration assay, multiple transgenic labels, regeneration inhibitor treatment and rescue)! Summary Fig at end of Fig 6 is excellent.

We are grateful to the Reviewer for their careful review of our study and that they find our work "novel" and has "implications for humans with defective ALDH2".
We thank the Reviewer for appreciating that we have tried to have careful presentation, but we take on board their comments that the writing is dense in places. In addition to the helpful comments from the Reviewers overall, we have reviewed the manuscript as a whole and tried to clarify and simplify where possible. All new text in the revision is indicated in blue.

My key comments are as follows:

1. Line (l) 75. Melanocyte regeneration is described as 'delayed', but authors do not show that there is further recovery with time, leaving open the question of whether it is 'delayed' or 'partially inhibited'?

We apologise for the confusion here, which was also spotted by another reviewer. Upon re-reading our manuscript we appreciate this was not clear because we had placed this data in **Figure S4A**. To address this comment and a similar comment above, we have repeated these experiments to get quantitative data (we previously only showed representative images) and placed this data in **Figure S1**. Our data shows that by 72 hours post washout – 8dpf – melanocyte regeneration in the *mitfa*^{vc7} had recovered in Aldh2i-treated embryos. We hope it is now easier for the reader to appreciate that Aldh2 inhibition causes a reversible delay in melanocyte regeneration.

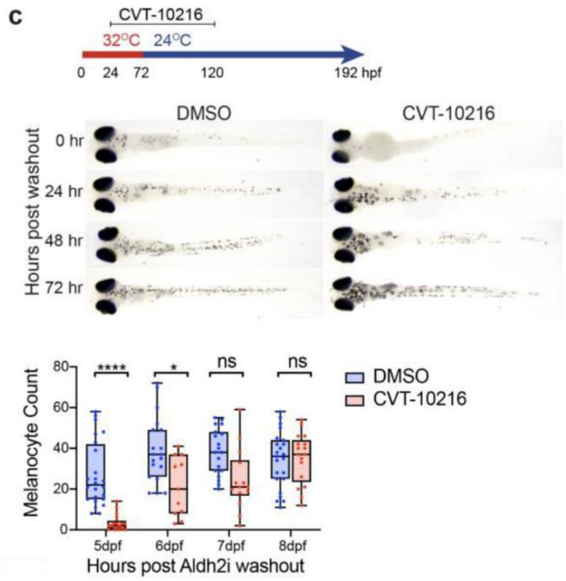

© 2022. Published by The Company of Biologists under the terms of the Creative Commons Attribution License (https://creativecommons.org/licenses/by/4.0/).

New Figure S1C: Extended regeneration assay on *mitfa*^{vc7} embryos treated with CVT-10216 from 24-120hpf. After washout, larvae were imaged, and melanocytes quantified at over time to monitor recovery/continuation of melanocyte regeneration. Representative images are shown from >5 embryos per condition, N=3. *: p<0.0332, ****: p<0.0001, ns: not significant. Kruskal-Wallis test with Dunn's multiple comparisons.

2. l.118 Assertion that McSCs re-express a NC identity seems to be based upon 1 marker, the crestin transgenic reporter. The authors should assess other markers, or tone down claim slightly.

Thank you for bringing this to our attention. We have now removed the work 'identity' and changed this sentence:

Thus, McSCs re-express a neural crest marker during regeneration and require Aldh2 to increase expression of mitfa and generate melanoblasts.

However, we do feel that our later scRNA-seq data shows McSCs have a neural crest identity. To clarify this, we have added FeaturePlots of other example early NC genes known to be expressed across multiple NC lineages to **Fig. S3**. *Foxd3* and *sox10* are expressed across multiple clusters, similar to *crestin*. Furthermore, *snai1b* and *pax3a* are expressed over multiple pigment clusters, including *crestin+ mitfa*-low McSCs.

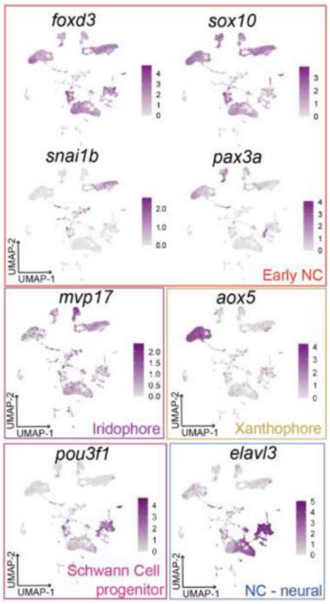

***New Figure S3**: UMAP of the combined dataset showing gene expression of early neural crest markers, and non-pigment clusters marked by pou3f1 (Schwann Cell Progenitors), mcamb and elavl3 marking NC-derived neural cells, mvp17 marking iridophores, and aox5 marking xanthophores.*

3. L158 onwards. Experimental rationale for this scRNA-seq experiment and its interpretation needs to be clearer. This whole section is v dense and thus made unnecessarily obscure to the reader. As I understand it, the crestin transgene will be labelling most/all NC derivatives, whereas the mitfa:GFP will label only the melanocytes, including regenerating ones, plus the activated McSCs from which regenerated melanocytes derive. Embryos were treated with either carrier (DMSO; control) or the Aldh2 inhibitor (in which the regeneration process is blocked downstream of activation of McSCs). But later, text refers to 'regenerating pigment clusters inc. melanoblasts'. It needs to be stated explicitly which are these? And do these include xanthophore and iridophore lineages (seem to be high in Aldh2)? If so, why are these regenerating after MoTP treatment? Indeed, what is evidence they are regenerating, rather than simply being labelled by crestin transgene. I think it would help if authors could add new panels to indicate which cell clusters were single and double labelled for transgenes….as it is, text seems to imply that they have been deduced indirectly from expression patterns (l. 175-180). Why is that necessary?

© 2022. Published by The Company of Biologists under the terms of the Creative Commons Attribution License (https://creativecommons.org/licenses/by/4.0/).

We thank the reviewer for this feedback and apologise for the confusion. We did not intend to assert that xanthophores and iridophores were also regenerating, and have now amended the text to read:
*aldh2.2 and aldh2.1 were expressed across multiple pigment cell clusters including McSCs and melanoblasts **(Fig. 4C)**.*

Thank you for the suggestion to add more panels indicating singly or doubly transgene-labelled cells. We now display this as part of **Fig. 4D**, which shows that cells in clusters 2,6 and 12 express predominantly *mCherry* but low *GFP* (*crestin+ mitfa*-low) whereas clusters 7 and 11 express a mixture of *GFP+* and *double+* cells (*crestin+ mitfa*-high).

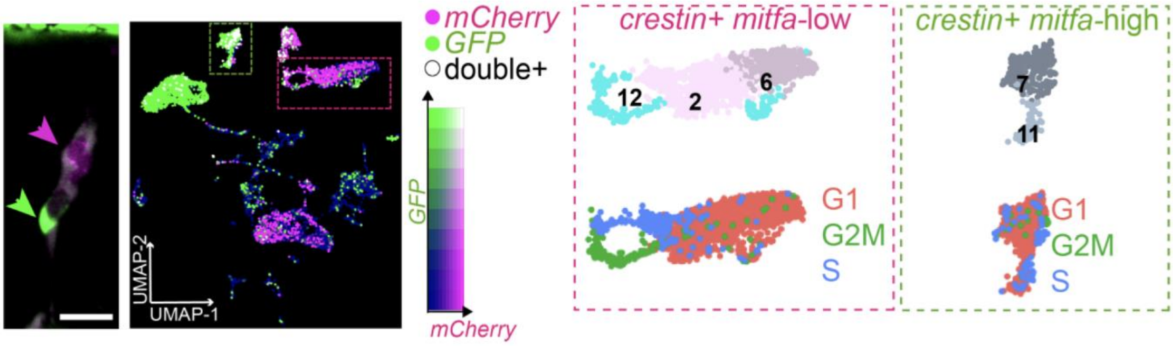

***New Figure 4D**: Proposed relation of imaged McSCs to scRNA-seq clusters, using an example niche from **Fig. 2D** (scale bar 20 μm) and UMAP coloured by expression intensity of mCherry (magenta), GFP (green) and cells in which both are expressed (white). We predict crestin+ mitfa-high cells (green arrow/box) are represented in clusters 7, 11, and crestin+ mitfa-low cells (magenta arrow/box) are represented in clusters 2,6,12. UMAPs of these clusters (top) and their predicted cell cycle phase (bottom) are shown.*

4. L184+ Needs to stated explicitly whether the higher proportion of crestin+/mitf- high cells is directly assessed, or simply deduced from size of clusters assigned (in previous paragraph) to these populations. It is currently unclear what is observation and what is inference.

We apologise for not making this clearer. We have amended the text to clarify that for each cluster, the numbers of cells that comprised it was expressed as a percentage of the total cells captured for each treatment condition. We present the analysis in this way because direct comparison of cell number within each cluster would have been misleading, given differences in total cell numbers between the two conditions.

*Next, we analyzed the dataset by drug treatment condition. Overall, we found that Aldh2 inhibition did not substantially change cell or cluster identity **(Fig. 4B)**. However, when comparing the numbers of cells within each cluster as a percentage of the total cell number per treatment condition, the proportions of cells within some clusters differed significantly **(Fig. 4E)**. Specifically, we detected a higher proportion of crestin+ mitfa-low cells (clusters 2,6,12), and a lower proportion of crestin+ mitfa-high cells (cluster 7) after ALDH2i.*

5. L198 Fig. S3d needs explanation for non-expert

Thank you for drawing this to our attention. We have included a further explanation in the Figure legend for **Figure S3D**, and labelled the axes further, which we hope clarifies this plot:

Figure S3D: GSEA enrichment plot of the de novo purine biosynthesis signature upregulated in clusters 2,6,12 in CVT-10216 treated embryos compared to control, generated using the Deseq2 output ranked by the Deseq2 test statistic. The green line and y-axis represent the enrichment score of the pathway, that is, the extent of correlation between the DMSO dataset to de novo purine synthesis relative to the Aldh2i dataset. Individual genes within this pathway are represented as vertical black lines, with genes contributing most towards the result - NES -1.18, FDR <25% (Kolganov Smirnov test) - boxed and listed to the right.

© 2022. Published by The Company of Biologists under the terms of the Creative Commons Attribution License (https://creativecommons.org/licenses/by/4.0/).

6. Fig. 1b Arrows indicating McSCs with low GFP not visible

   We thank the Reviewer for this comment but feel there might be a misunderstanding. The purpose of this image was to introduce the reader to what an establishing McSC niche looked like in terms of its location relative to the DRG and neurons (labelled with the *nbt:dsRed* transgene) as opposed to embryonic melanocytes settling into the epidermis. If there are low *GFP*-expressing cells in this niche, it would be difficult to see them without the *crestin:mCherry* transgene.

7. Fig. 4. How many cell profiles (post-QC) form the dataset for the clustering analysis? (This buried in M&M but would be good to know here)
   Thank you for this excellent suggestion. We have clarified this in the **Main Text** and added more detail on the suggestion of another Reviewer, so the text now reads:

   *To this end, we designed a scRNA-seq analysis of a MoTP melanocyte regeneration experiment in which double transgenic mitfa:GFP; crestin:mCherry embryos were treated with DMSO or CVT-10216, and then GFP+, mCherry+ and double+ cells sorted together by FACS and processed for 10x sequencing (**Fig. 4A**). This yielded 4488 DMSO-treated cells and 6795 Aldh2i-treated cells, which were aggregated into a single dataset for cluster identification. We identified 24 clusters of transcriptionally distinct cell populations...*

8. Fig. 5c States that single channel images are shown alongside merges, but all look merged to me. Consequently hard to assess claim that crestin+mitfa-low cells unchanged by formate treatment.
   We thank the Reviewer for this helpful comment and apologise for not spotting this before submitting. We have now re-instated both the single channel and merged images and hope that this now better supports our assertion that *crestin+ mitfa-low* cells are unaffected by formate supplementation.

   Minor points:

   In general, I found the figure legends to be rather minimal and in some cases figures take a lot of studying to decipher. This could be improved by explicit explanation of all features shown. In some cases the deficiencies were more persistent:
   We apologise for the somewhat sparse nature of some of the legends. This was chiefly due to most figures having a lot of panels and a word limit for each legend of 300 words. We have addressed the specific comments below, and hope this is helpful for clarity.

9. Supp. Fig. 1 Generation of aldh2-/-. Authors need to clarify location of both primers in Primer pairs 1 and 2. How do these relate to the small blue and green arrows (if at all)?

   We apologise for the confusion here. We did not show the exact positions of both forward and reverse RT-qPCR primers to this schematic, as the ~120bp they cover in relation to the whole gene is very small. We have expanded upon this in the **Methods**.

   *RT-qPCR primers (**Table S7**) were designed using Primer 3 Plus software to amplify ~120bp regions over exon-intron junctions. In the case of using RT-qPCR to detect aldh2-/- mutant transcripts, these regions were picked to be either in a predicted region of aldh2.1 still present after the fusion event (Primer site 1, exon 5 of aldh2.1, **Fig. S1**), or within a region of aldh2.2 predicted to disappear after excision of the intergenic region between aldh2.1 and aldh2.2 (Primer site 2, exon 13 of aldh2.2).*

   During this revision time we took the opportunity to add a western blot using an ALDH2 antibody to show that there is no Aldh2 protein made in mutant embryos (**Fig. S1**).

   The new Figure panel and legend read as follows:

© 2022. Published by The Company of Biologists under the terms of the Creative Commons Attribution License (https://creativecommons.org/licenses/by/4.0/).

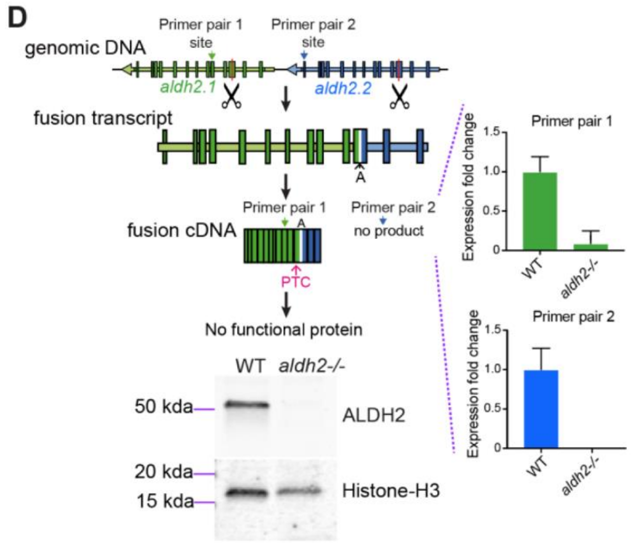

***Figure S1D**: sgRNAs targeting exon 3 of aldh2.1 and aldh2.2 were co-injected with Cas9, causing deletion of the intergenic region and creation of a fusion transcript, containing a 1 bp insertion and premature termination codon (PTC). RT-qPCR was performed with primers targeting Primer site 1, persisting in the truncated fusion transcript, and Primer site 2 (excised), see Methods. Housekeeping control: β-actin. 3 experimental replicates. Error bars represent s.e.m. Western blot analysis of ALDH2 protein in aldh2-/- and WT embryos confirms a lack of Aldh2 protein in mutants. Loading control: Histone-H3.*

10. Supp. Fig. 3d. This needs a brief explanation for those unfamiliar with the approach. What is plotted on x axis?
Thank you for this comment. We have now expanded the figure legend for this plot which we hope clarifies it (please see response to comment 5 above).

References

Brombin A, Simpson DJ, Travnickova J, Brunsdon H, Zeng Z, Lu Y, Young AIJ, Chandra T, Patton EE. 2022. Tfap2b specifies an embryonic melanocyte stem cell that retains adult multifate potential. *Cell Rep* **38**: 110234.

Budi EH, Patterson LB, Parichy DM. 2008. Embryonic requirements for ErbB signaling in neural crest development and adult pigment pattern formation. *Development* **135**: 2603-2614.

-. 2011. Post-embryonic nerve-associated precursors to adult pigment cells: genetic requirements and dynamics of morphogenesis and differentiation. *PLoS Genet* **7**: e1002044.

Chen CH, Ferreira JC, Gross ER, Mochly-Rosen D. 2014. Targeting aldehyde dehydrogenase 2: new therapeutic opportunities. *Physiol Rev* **94**: 1-34.

Dooley CM, Mongera A, Walderich B, Nusslein-Volhard C. 2013. On the embryonic origin of adult melanophores: the role of ErbB and Kit signalling in establishing melanophore stem cells in zebrafish. *Development* **140**: 1003-1013.

El-Brolosy MA, Kontarakis Z, Rossi A, Kuenne C, Gunther S, Fukuda N, Kikhi K, Boezio GLM, Takacs CM, Lai SL et al. 2019. Genetic compensation triggered by mutant mRNA degradation. *Nature* **568**: 193-197.

Farnsworth DR, Saunders LM, Miller AC. 2020. A single-cell transcriptome atlas for zebrafish development. *Dev Biol* **459**: 100-108.

Johnson SL, Nguyen AN, Lister JA. 2011. mitfa is required at multiple stages of melanocyte differentiation but not to establish the melanocyte stem cell. *Dev Biol* **350**: 405-413.

Kaufman CK, Mosimann C, Fan ZP, Yang S, Thomas AJ, Ablain J, Tan JL, Fogley RD, van Rooijen E, Hagedorn EJ et al. 2016. A zebrafish melanoma model reveals emergence of neural crest identity during melanoma initiation. *Science* **351**: aad2197.

Saunders LM, Mishra AK, Aman AJ, Lewis VM, Toomey MB, Packer JS, Qiu X, McFaline-Figueroa JL, Corbo JC, Trapnell C et al. 2019. Thyroid hormone regulates distinct paths to maturation in pigment cell lineages. *Elife* **8**.

© 2022. Published by The Company of Biologists under the terms of the Creative Commons Attribution License (https://creativecommons.org/licenses/by/4.0/).

Singh AP, Dinwiddie A, Mahalwar P, Schach U, Linker C, Irion U, Nusslein-Volhard C. 2016. Pigment Cell Progenitors in Zebrafish Remain Multipotent through Metamorphosis. *Dev Cell* **38**: 316-330.

Second decision letter

MS ID#: DEVELOP/2021/200277

MS TITLE: Aldh2 is a lineage-specific metabolic gatekeeper in melanocyte stem cells

AUTHORS: Hannah Brunsdon, Alessandro Brombin, Samuel Peterson, John H Postlethwait, and E. Elizabeth Patton
ARTICLE TYPE: Research Article

Sorry for the delay in making a decision on your manuscript. However, I am happy to tell you that the referees are happy with your revisions and your manuscript has been accepted for publication in Development, pending our standard ethics checks. The referee reports on this version are appended below.

Reviewer 1

Advance summary and potential significance to field

My issues in the first submission have been satisfactorily addressed in the revised one.

Comments for the author

no additional suggestions

Reviewer 2

Advance summary and potential significance to field

In this manuscript, Brunsdon et al. define the role of Aldh2-dependent formaldehyde metabolism in melanocyte stem cell (McSC) regeneration in zebrafish.
Using melanocyte regeneration assays coupled with scRNAseq and live fluorescent imaging of McSC reservoirs in the DRG, the authors show that 1) Aldh2 is required for McSC regeneration, promoting increased mitfa expression and progeny formation and migration, 2) McSC regeneration relies on Aldh2 to catalyze the metabolism of formaldehyde to formate, and 3) Purine synthesis through the 1C cycle is required for McSC regeneration. The authors also show that McSC regeneration involves re-activation of neural crest identity, but that Aldh2-dependent metabolism drives an increase of mitfa expression that is required for melanoblast formation.
Together, this work mechanistically describes a fundamental process required for melanocyte differentiation and migration. This publication will therefore contribute much to the field of development and stem cell biology.

Comments for the author

In this revised manuscript, Brunsdon et al. have provided sufficient revisions for each of our reviewer comments. This manuscript will contribute much to the field of melanocyte development and will benefit the readers of Development. We recommend it for publication.

© 2022. Published by The Company of Biologists under the terms of the Creative Commons Attribution License (https://creativecommons.org/licenses/by/4.0/).

Reviewer 3

Advance summary and potential significance to field

The authors have worked hard to address my concerns, and have done so successfully.

Comments for the author

Thansk for addressing thoroughly all the queries raised.

© 2022. Published by The Company of Biologists under the terms of the Creative Commons Attribution License (https://creativecommons.org/licenses/by/4.0/).
